# The FAD-dependent glycerol-3-phosphate dehydrogenase of *Giardia duodenalis:* an unconventional enzyme that interacts with the g14-3-3 and it is a target of the antitumoral compound NBDHEX

**DOI:** 10.3389/fmicb.2015.00544

**Published:** 2015-06-01

**Authors:** Marco Lalle, Serena Camerini, Serena Cecchetti, Renata Finelli, Gabriella Sferra, Joachim Müller, Giorgio Ricci, Edoardo Pozio

**Affiliations:** ^1^Department of Infectious, Parasitic and Immunomediated Diseases, Istituto Superiore di SanitàRome, Italy; ^2^Department of Cell Biology and Neurosciences, Istituto Superiore di SanitàRome, Italy; ^3^Institute of Parasitology, Vetsuisse Faculty, University of BernBern, Switzerland; ^4^Department of Sciences and Chemical Technologies, University of Rome “Tor Vergata”Rome, Italy

**Keywords:** *Giardia duodenalis*, FAD-dependent glycerol-3-phoshate dehydrogenase, 14-3-3 protein, energy metabolism, mitosome, encystation, NBDHEX, nitroreduction

## Abstract

The flagellated protozoan *Giardia duodenalis* is a worldwide parasite causing giardiasis, an acute and chronic diarrheal disease. Metabolism in *G. duodenalis* has a limited complexity thus making metabolic enzymes ideal targets for drug development. However, only few metabolic pathways (i.e., carbohydrates) have been described so far. Recently, the parasite homolog of the mitochondrial-like glycerol-3-phosphate dehydrogenase (gG3PD) has been identified among the interactors of the g14-3-3 protein. G3PD is involved in glycolysis, electron transport, glycerophospholipids metabolism, and hyperosmotic stress response, and is emerging as promising target in tumor treatment. In this work, we demonstrate that gG3PD is a functional flavoenzyme able to convert glycerol-3-phosphate into dihydroxyacetone phosphate and that its activity and the intracellular glycerol level increase during encystation. Taking advantage of co-immunoprecipitation assays and deletion mutants, we provide evidence that gG3PD and g14-3-3 interact at the trophozoite stage, the intracellular localization of gG3PD is stage dependent and it partially co-localizes with mitosomes during cyst development. Finally, we demonstrate that the gG3PD activity is affected by the antitumoral compound 6-(7-nitro-2,1,3-benzoxadiazol-4-ylthio)hexanol, that results more effective *in vitro* at killing *G. duodenalis* trophozoites than the reference drug metronidazole. Overall, our results highlight the involvement of gG3PD in processes crucial for the parasite survival thus proposing this enzyme as target for novel antigiardial interventions.

## Introduction

The flagellated protozoan *Giardia duodenalis* (syn. *lamblia*, *intestinalis*) is a parasite of the upper part of small intestine of mammals, including humans. Infection with *G. duodenalis* causes giardiasis, one of the most common foodborne and waterborne gastroenteric diseases (Halliez and Buret, 2013; Ryan and Cacciò, 2013). The parasite has a simple two-stages life cycle consisting of the trophozoite, that replicates and colonizes the host intestine causing symptoms, and the cyst, the environmentally resistant stage that is spread with feces and is responsible for transmission of the infection. Generally, infection is acquired by ingestion of cysts in contaminated water and food or by the fecal–oral route (Ryan and Cacciò, 2013). Clinical symptoms of giardiasis can vary from asymptomatic infection to acute and chronic diarrhea, ultimately leading to chronic post-infectious gastrointestinal complications, including irritable bowel syndrome and chronic fatigue (Halliez and Buret, 2013). Up to date, no human vaccine for giardiasis is available and treatment relies only on a limited panel of effective approved drugs. Nitroheterocyclics, such as metronidazole (MTZ) and nitazoxanide (NTZ), are the antigiardial drugs of choice. Unfortunately, treatment failure has been reported in 10–20% of cases and strains resistant to different compounds have been either clinically isolated or induced *in vitro* (Lalle, 2010; Watkins and Eckmann, 2014). In this scenario, alternative, safe, and effective therapies are required.

*Giardia duodenalis* has a peculiar energy metabolism. It is a microaerophilic organism that, instead of mitochondria, contains mitosomes, highly reduced mitochondria-derived organelles which sole function seems to be restricted to Fe-S cluster biosynthesis (Tovar et al., 2003; Jedelský et al., 2011). Energy is then generated by substrate level phosphorylation and fermentation occurring in the cytoplasm or at the inner side of the plasma membrane (Adam, 2001). In terms of sequence similarity, these metabolic pathways of *G. duodenalis* consist of a mixture of eukaryote-like and bacteria-like enzymes. Therefore, energy and intermediate metabolism of *G. duodenalis* have been shown to provide opportunities to identify novel and effective compounds, as well as potentially interesting targets by means of high-throughput drug screening and target-based drug design (Müller and Hemphill, 2013; Watkins and Eckmann, 2014).

We recently detected a putative glycerol-3-phosphate dehydrogenase/flavin-dependent oxidoreductase (gG3PD, GL50803_16125) of *G. duodenalis* among proteins interacting with the parasite 14-3-3 isoform, g14-3-3 (Lalle et al., 2012). The 14-3-3s are a family of highly conserved eukaryotic phosphoserine/phosphothreonine-binding proteins which participate to the regulation of key cellular processes by direct interaction with 100s of target proteins (Gardino and Yaffe, 2011; Kleppe et al., 2011). The characterized g14-3-3 interactome provides evidences that g14-3-3 can be involved in parasite energy metabolism, as supported by the identification and confirmation of components of both the glycolytic/gluconeogenetic pathway and pyruvate metabolism (Lalle et al., 2012).

Flavin adenine dinucleotide (FAD)-dependent glycerol-3-phosphate dehydrogenase (G3PD, EC 1.1.5.3) is a key enzyme at the crossroad of glycolysis, redox, and fatty acid metabolism, in both prokaryotes and eukaryotes. G3PD catalyzes the oxidation of glycerol-3-phosphate (G3P) to dihydroxyaceton phosphate (DHAP) with simultaneous reduction of FAD to FADH_2_ and transfer of electrons to quinones (e.g., ubiquinone; Unden and Bongaerts, 1997; Mráček et al., 2013). In eukaryotes, a single subunit enzyme (mG3PD, 69–75 kDa) is strongly associated, as peripheral protein, with the outer face of the inner mitochondrial membrane (Janssen et al., 2002; Mráček et al., 2013). The mG3PD has multiple functions: (i) it forms the glycerophosphate shuttle in combination with the cytosolic NADH-dependent G3PD (cG3PD, EC 1.1.1.8) to re-oxidize the cytosolic NADH produced by glycolysis; (ii) it is part of the mitochondrial respiratory electron transport chain (ETC) channeling electron to quinone pool and bypassing Complex I; (iii) it regulates the cytosolic level of G3P (Bell and Coleman, 1980; Mráček et al., 2013). Prokaryotes harbor two distinct membrane-associated FAD-dependent G3PDs, both necessary for bacterial growth in presence of glycerol or G3P as sole carbon source, and represent key primary dehydrogenases transferring reducing equivalents to a short respiratory ETC with different terminal reductases and electron acceptor (O_2_, nitrate, or fumarate; Unden and Bongaerts, 1997). The homodimeric GlpD is associated with the cytoplasmic side of plasma membrane, shows 30–33% homology with eukaryotic mG3PD, and is expressed under aerobic conditions when O_2_ is the terminal acceptor (Walz et al., 2002; Yeh et al., 2008). The heterotrimeric GlpACB is induced under anaerobic conditions, with fumarate as terminal acceptor (Cole et al., 1988; Varga and Weiner, 1995): it forms a functional-associated complex with fumarate reductase and contributes to generate a proton gradient across the membrane via an associated ETC (Miki and Wilson, 1978). The GlpAC heterodimer (62 and 41 kDa, respectively) is the soluble catalytic subunit, containing the FAD and FMN binding sites (Cole et al., 1988). The GlpB (44 kDa) subunit, that contains a ferredoxin-type (4Fe-4S) cluster binding motif, anchors the GlpAC dimer to the inner cytoplasmic membrane and mediate the transfer of reducing equivalent to the menaquinone pool and finally to the fumarate reductase (Varga and Weiner, 1995).

The G3PDs are also involved in pathogenicity as a source of reactive oxygen species (ROS). The mG3PD has been implied in the establishment of a pro-oxidative environment that promotes the fast growth of undifferentiated tumors (Chowdhury et al., 2007; Mráček et al., 2013), whereas the oxidase activity of GlpD of *Mycoplasma* sp. seems to be crucial for the pathogenicity, leading to high levels of H_2_O_2_, and host cell damage (Großhennig et al., 2013).

In the present work, we investigated gG3PD expression, activity, and cellular localization in the *G. duodenalis* trophozoite stage and during the encystation process. Moreover, we demonstrated that the antitumoral compound [NBDHEX, 6-(7-nitro-2,1,3-benzoxadiazol-4-ylthio)hexanol] is an effective antigiardial compound that negatively affected gG3PD activity. Overall, our results pointed out the role of gG3PD in biological processes crucial for the parasite survival, thus suggesting that this enzyme could represent a good candidate for targeted antigiardial interventions.

## Materials and Methods

### Chemicals

The NBDHEX was synthetized as previously described (Ricci et al., 2005). MTZ, FAD disodium salt, sn-g3p, phenazine methosulfate (PMS), 3-(4,5-dimethylthiazol-2-yl)-2,5-diphenyltetrazolium bromide (MTT) were from Sigma–Aldrich (St. Louis, MO, USA).

### Parasite Cultivation, Differentiation, and Transfection

The *G. duodenalis* isolate WB clone C6 (WB-C6) was axenically grown in TYI-S-33 medium at 37°C and differentiation into cyst (encystation) was induced in TYI-S-33 medium containing 5 mg/ml of bovine bile at pH 7.8 for the indicated time (Schupp et al., 1988). Parasites were collected by chilling tubes on ice and centrifugation at 800 ×*g*. Transgenic lines were generated by trophozoite electroporation with 15 μg of plasmid DNA and selected and maintained under constant selection with 100 μM puromycin (Invivogen, Toulouse, France).

### Vector Construction

*Escherichia coli* BL21-DE3 competent cells were used. The full-length sequence of gG3PD (accession number GL50803_16125), the gG3PD N-terminal (nucleotide 3-1590; gG3PD_N), and the C-terminal half (nucleotide 1569-3333; gG3PD_C), were PCR amplified from the *G. duodenalis* WB-C6 genomic DNA, prepared as previously described (Lalle et al., 2012). The primers used and their combinations are reported in **Table 1**. Reactions were performed on a T-Personal Thermocycler (Biometra, Göttingen, Germany) using 100 ng of gDNA, 10 units of high fidelity Pfu turbo DNA polymerase (Agilent Technologies, Santa Clara, CA, USA), 50 μM dNTP, 20 pmols of each primer in 50 μl of reaction mixture. Amplification conditions were: 1 cycle at 95°C for 2 min; 30 cycles at 95°C for 30 s, 58°C for 30 s, and 72°C for 30 s; and 1 cycle at 72°C for 7 min. PCR fragments were cloned either in the *BamHI/PspOMI* digested pTUB-FLAG_HApac vector (Lalle et al., 2012) for expression in *G. duodenalis*, or in the *BamHI/NotI* digested pQ30 vector (Qiagen, Germany) for expression in bacteria as N-terminal 6xHIS-tagged fusion protein.

**Table 1:** Primer list.

Target	Forward primer^a^	Reverse primer^b^
Full length glycerol-3-phosphate dehydrogenase (gG3PD)	**gG3PDforw** 5′-GGATCCACCACCGCTCACCCTTTACC-3′	**gG3PDrev** 5′-GCGGCCGCTCACTTCATAGCCGGAATGTTC-3′
N-terminal gG3PD	**gG3PDforw**	**NTrev** 5′-GCGGCCGCTCAGTATGCCTTGTCCATCGGGGTG-3′
C-terminal gG3PD	**CTforw** 5′-GGATCCACCCCGATGGACAAGGCATAC-3′	**gG3PDrev**

^a^The BamHI site is underlined.

^b^The NotI site is underlined.

### Expression and Purification of the Recombinant Proteins

Transformed *E. coli* were grown in SOB medium and recombinant proteins expression was induced at OD_600_ = 0.6, with 0.5 mM isopropyl-thio-β-D-galactoside at 37°C for 4 h. All 6xHIS-fused proteins were purified under native condition by affinity chromatography on nickel resin (Qiagen) and eluted with 250 mM imidazole (pH 8.0). Proteins were dialyzed against PBS (140 mM NaCl, 2.7 mM KCl, 10 mM Na_2_HPO_4_, 1.8 mM KH_2_PO_4_, pH 7.4) using Slide-A-Lyzer dialysis cassettes (cut-off 3.5 kDa, Thermo Fisher Scientific, Rockford, IL, USA). The protein concentration was determined by Bradford assay (Thermo Fisher Scientific) and proteins were stored at -70°C until use.

### Production of Polyclonal Antibodies

Purified HIS-gG3PD fusion protein was used to immunize intraperitoneally two BALB/c mice (Charles River Laboratories International, Inc., Wilmington, MA, USA) on days 0, 21, and 42 with 50 μg protein in 300 μl of emulsified 1:1 PBS/Freund’s complete adjuvant (Sigma–Aldrich; at day 0) or 1:1 PBS/Freund’s incomplete adjuvant (Sigma–Aldrich; at day 21) or without any adjuvant (at day 42). Blood was collected from the tail vein before initial immunization and after each boost. Sera fractions were assayed for specific antibody content.

### Preparation of *G. duodenalis* Proteins

Total soluble proteins (S) and octyl β-D-glucopyranoside solubilized proteins from membranous material (M) were prepared as previously described (Lalle et al., 2012) starting from 2 × 10^9^ trophozoites or encysting parasites. The protein concentration was determined by Bradford methods (Thermo Fisher Scientific) and the protein lysates were stored at -70°C. Alternatively, to assess the G3PD enzymatic activity in *G. duodenalis*, 10^6^ parasites were collected, washed two times with cold PBS, suspended in 200 μl of PBS/1% TritonX100 and incubated 1 h on ice. After centrifugation at 13.000 ×*g* for 15 min, supernatant was collected, protein concentration quantified by Bradford methods and the preparation was immediately used for the enzymatic assay as detailed below.

### Western Blot Analysis

Proteins were separated on SDS-PAGE and transferred onto PVDF membrane with 39 mM glycine, 48 mM Tris, 0.1% SDS, and 10% methanol, using a semidry apparatus (BioRad, Hercules, CA, USA). Membranes were blocked with 5% skin milk in TTBS (20 mM Tris-HCl, pH 7.5, 100 mM NaCl, 0.05% Tween 20) for 1 h and then incubated with the primary antibody (Ab) in 2.5% skin milk/TTBS buffer. After incubation with an appropriate HRP-conjugated secondary Ab (1:3000), the interaction was revealed by chemiluminescence (Millipore, France). Antibodies were used at the following dilution: mouse polyclonal anti-gG3PD antiserum 1:3000; mouse anti-HA mAb (Sigma–Aldrich) 1:3000; rabbit N14 (anti-g14-3-3) antiserum (Lalle et al., 2006) 1:5000; mouse anti-αTubulin (Sigma–Aldrich) 1:10000; rabbit anti-gPGN (phosphoacetylglucosamine mutase, Lopez et al., 2003) 1:1000; mouse anti-HIS mAb (Qiagen) 1:2000.

### Blue Native PAGE (BN-PAGE)

Three to twelve percentage of BN-PAGE (Invitrogen, Carlsbad, CA, USA) was carried out using 1 μg of purified HIS-tagged recombinant proteins according to the manufacturer. This technique allows the separation of very high molecular weight multiprotein complexes. Gels were run in Running buffer (0.002% Coomassie G-250, 50 mM BisTris/50 mM Tricine, pH 6.8) at 150 V for approximately 2 h. Gels were incubated in Tris/glycine/SDS buffer for 30 min and either stained with a Silver Staining kit (Invitrogen) or processed for western blot as described.

### Affinity Purification

FLAG-tagged proteins were purified by affinity chromatography on mouse anti-FLAG M2 mAb covalently bound to agarose beads (Sigma–Aldrich) as previously reported (Lalle et al., 2012) and directly eluted from the resin by incubation with 200 μM FLAG-peptide at 4°C for 1 h. The eluted materials were stored at -70°C until use.

### Confocal Laser Scanning Microscopy (CLSM)

Trophozoites or encysting parasites were fixed and permeabilized as previously described (Lalle et al., 2006). Antibodies were used at the following dilution: polyclonal rabbit N14 antiserum 1:50; mouse polyclonal anti-gG3PD antiserum 1:50; polyclonal rabbit anti-gTom40 antiserum (Dagley et al., 2009) 1:50, FITC-conjugated mouse anti-HA mAb (Miltenyi Biotec, Germany) 1:50; Cy5-conjugated anti-CWP mAb (Waterborne Inc., New Orleans, LA, USA) 1:30. Alexa-Fluor 647- and 488-conjugated anti-rabbit and anti-mouse secondary Ab (Invitrogen) were used at a 1:500 dilution. After parasite staining, coverslips were extensively rinsed and then mounted using Vectashield^®^ mounting medium (Vector Laboratories Inc., Burlingame, CA, USA) containing 300 nM of 4′,6-diamidino-2-phenylindole (DAPI). Confocal laser scanning microscopy (CLSM) observations were performed on a Leica TCS SP2 AOBS (Leica Microsystems, Germany) apparatus, using excitation spectral laser lines at 405, 488, and 647 nm and selecting emission wavelengths by a proper setting of the spectral detection system. Signals from different fluorescent probes were taken in sequential scan mode. Image acquisition and processing were conducted using the Leica Confocal Software (Leica Microsystems). Image deconvolution was performed using the Huygens software (Scientific Volume Imaging BV, Hilversum, The Netherlands). Data were inferred from two independent experiments following the analysis of different fields of view (>200 cells) on the microscope for each labeling condition.

### G3PD Enzymatic Assay, Spectrophotometric, and Fluorimetric Analysis

The dehydrogenase activity of gG3PD was assayed spectrophotometrically according to Kistler and Lin (1972) measuring the rate of PMS-mediated reduction of the tetrazolium dye, MTT, to its formazan by addition of g3p. Reactions were performed in 96 well plate using either 100 μg of *G. duodenalis* Triton X-100 protein lysate or 16 pmol of purified recombinant HIS-tagged proteins in a final volume of 200 μl containing 67 mM of potassium phosphate (pH 7.5), 0.2% Triton X100, 6.6 μg of MTT, 20 μg of PMS, and 17 mM of g3p with or without 10 μM FAD. The g3p was omitted in the blank. Since crude extracts could contain substantial endogenous substrates, to ensure a linear rate of MTT reduction, reactions were pre-incubated at 25°C for 5 min prior to the addition of MTT and g3p. Plates were sealed with an air-tight adhesive tape (Greiner Bio One, Austria) to prevent evaporation. After blank subtraction, the enzyme activity was calculated and expressed as micromoles per minute per milligram of protein, considering that the extinction coefficient of reduced MTT is 17 mM^-10^cm^-1^ a 570 nm. UV-visible spectra of HIS-gG3PD (10 μg/μl) in 67 mM of potassium phosphate (pH 7.5), were recorded at 25°C in 10 μl quartz capillaries. UV-visible spectra of 100 μM NBDHEX were recorded in 67 mM of potassium phosphate (pH 7.5), 17 mM of g3p, with or without HIS-gG3PD (42 pmol) in 0.5 ml quartz cuvette. All measures were done at 25°C in a Multiskan Spectrum (Thermo) spectrophotometer. Fluorescent spectra (excitation at 430 nm) were acquired in a Luminescence Spectrometer LS50B (Perkin–Elmer, Waltham, MA, USA).

### Determination of Intracellular Glycerol

For the assay, 10^6^ parasites were collected as previously described, suspended in 100 μl of PBS, incubated 10 min at 95°C and the supernatant collected after centrifugation at 12,000 ×*g* for 1 min. Protein concentration was measured by Bradford assay. Intracellular glycerol level was enzymatically determined using the Free Glycerol Reagent kit (Sigma–Aldrich) by measuring spectrophotometrically the production of a quinoneimine dye at 540 nm. Assay was performed on 96 well-plate according to the manufacturer’s instruction, using 40 μl of parasite lysate and 160 μl of enzyme mixture per well. After 5 min of incubation at 37°C, absorbance was read by Multiskan Spectrum (Thermo Scientific) spectrophotometer. Intracellular glycerol concentration was calculated interpolating the obtained absorbance with a glycerol standard curve and expressed as pmol of glycerol/μg of total protein. Each condition was assayed in triplicate and the experiment was performed independently three times.

### *In Vitro* Drug Susceptibility Assay

The *in vitro* assays were performed according to Bénéré et al. (2007) and Hounkong et al. (2011) with modifications. All compounds were dissolved and serially diluted in 1:1vol ethanol/DMSO. *G. duodenalis* trophozoites (5 × 10^5^ parasite/ml) were cultured in 96-well plates (Nunc Δ surface, Thermo Scientific) in TYIS-33 medium and 3 μl of 100X concentrated compound, or solvent, were added to reach the desired compound concentration. The plates were sealed with an air-tight adhesive tape (Greiner Bio-One GmbH, Austria) and incubated for 48 h at 37°C. After the incubation period, culture medium was removed. Adherent trophozoites were immediately fixed with 300 μl of methanol for 2 min and then stained with a solution of 0.1% methylene blue in PBS for 10 min at RT. Wells were washed three times with PBS and incubated with 200 μl of ethanol and 0.1 M HCI [1:1 (v/v) in H_2_O], to elute the dye. Absorbance was determined at 655 nm by Multiskan Spectrum (Thermo Scientific) microplate spectrophotometer. Growth inhibition was calculated as the percentage of treated parasite in comparison with untreated parasite. Three independent experiments were performed and each drug dilution was assayed in triplicate. Alternatively, for drug efficacy tests with resazurin (Bénéré et al., 2007), trophozoites were grown in the presence of serial dilutions of the drugs, or DMSO as control, in an anaerobic growth chamber (100% N_2_, 37°C). After 72 h, the medium was removed, the wells were washed three times with pre-warmed PBS containing 1% of glucose (0.2 ml per well), and finally, 0.2 ml PBS with glucose containing 10 mg/l resazurin were added. Reduced resazurin was quantified by fluorimetry (excitation at 365 nm, emission at 455 nm) using a 96-well-multimode plate reader Enspire (Perkin–Elmer).

For immunolocalization and enzymatic assays, drug treatments were performed in 10 ml screw cap tubes using 1 × 10^5^ parasite/ml of *G. duodenalis* trophozoites. Twenty five microliter of 20 mM NBDHEX in ethanol, or the equal volume of ethanol, was added to 10 ml of TYS-I-33 medium (final concentration 50 μM) and parasites were incubated at 37°C for the indicated time periods and cells were processed as described in previous paragraphs.

### Mass Spectrometry Analysis

Proteins were separated on a 1D-gel NuPAGE 4-12% (Novex, Invitrogen) run in morpholinepropanesulfonic acid (MOPS) buffer and stained with the Colloidal Blue Staining kit (Invitrogen). Slices were excised and digested with modified sequencing-grade trypsin (Promega Corporation, France), as previously described (Shevchenko et al., 1996). Nanoflow reversed-phase liquid chromatography tandem mass spectrometry (RP-LC-MS/MS) analysis of peptide mixtures was performed using an HPLC Ultimate 3000 (DIONEX, Sunnyvale, CA, USA) coupled with a linear ion trap (LTQ, Thermo Electron, San Jose, CA, USA) mass spectrometer. Peptides were desalted in a trap column (Acclaim PepMap 100 C18, LC Packings, DIONEX) and separated in the analytical column, a 10 cm long fused silica capillary (Silica Tips FS 360-75-8, New Objective) in house slurry-packed with a 5 μm, 200 Å pore size C18 resin (Michrom BioResources, Auburn, CA, USA). Peptides were eluted using a 40 min linear gradient from 96% aqueous buffer containing 5% acetonitrile and 0.1% formic acid to 60% organic buffer constituted by acetonitrile with 5% H_2_O and 0.1% formic acid, at 300 nL/min flow rate. Analyses were performed in positive ion mode and the HV Potential was set up around 1.7–1.8 kV. Tandem mass spectra were matched against the *G. duodenalis* protein database (Giardia DB version 1.2) downloaded from the Web site http://www.giardiadb.org/giardiadb and through the SEQUEST algorithm incorporated in the Bioworks software (version 3.3, Thermo Electron). The following match parameters were considered: fully tryptic cleavage constraints (one miscleavage allowed), static cysteine carbamidomethylation, and variable methionine oxidation. Precursor and fragment ions were searched with 1.5 and 1 Da tolerance, respectively. Possible NBDHEX adducts on cysteine or lysine residues were searched. The mass increments considered were +296, +281, +265 Da, for the intact, partially or completely nitro-reduced NBDHEX adducts, respectively. Statistical parameters used for legitimate protein identification were described elsewhere (Lalle et al., 2012).

### Chemical Reduction of NBDHEX

Sodium dithionite was prepared as a 1M solution in ddH_2_O and was used 100 mM to chemically reduce 10 mM NBDHEX in 20 μl 0.5% NH_3_. After 5 min, 2 μl reaction was diluted to 200 μl in 67 mM potassium phosphate buffer (pH 7.5). NBDHEX fluorescence quenching was verified at 25°C in a Luminescence Spectrometer LS50B (Perkin–Elmer) whereas the UV-visible spectra was acquired at 25°C in a Multiskan Spectrum (Thermo Scientific) spectrophotometer in 0.5 ml quartz cuvettes. For the mass spectrometry analysis, the reaction mixture was diluted in 50% ethanol and 1% ammonia and directly infused in the LTQ mass spectrometer through a glass tip. The nanospray ionization in positive ion mode was allowed applying 1.5–1.6 kV. HV Potential. Full MS in the 150–350 m/z range were acquired; then MS3 fragmentation of the ions 280 and 250, derived from MS2 of 298 and 268, respectively, was induced.

### Sequence Analysis

Conserved functional domains and sites in the protein sequence were search using ELM (Dinkel et al., 2014) and BLASTP^1^ algorithms. Transmembrane (TM) regions were search by TMHMM Server v.2.0^2^. Mitochondrial targeting signal were search using Psort II^3^ and MitoProt II^4^. Genomic sequences of 1133 organisms were downloaded from EggNog database v.3.0 (Powell et al., 2012). If more than one strain was available, only the strain classified as “core” was retained. This selection resulted in 774 organisms, which were included in the reference set. The Smith–Waterman algorithm available in the FASTA package (Pearson and Lipman, 1988) was exploited using as query the gG3PD full-length sequence, or the protein portions encompassing: the first 500 residues (referred as N-terminal domain); or the residues 501–950 (referred as central domain); or the residues 951–1111 (referred as the C-terminal domain); or the protein portion including the N- and central domains (residues 1–950). Sequences having a match result with *E*-value lower than 10^-6^ were selected, except for the C-terminal domain for which the *E*-value threshold was set at 10^-3^. Sequence analysis, editing and phylogenetic analysis were performed using Bioedit (Hall, 1999), MEGA 5.0 (Tamura et al., 2013), and Jalview 2.8.2 (Waterhouse et al., 2009).

### Statistics

Statistical analyses were performed with PRISM 6.0 software (GraphPad Software, Inc; La Jolla, CA, USA) and significance was calculated by Student unpaired *t*-test and one-way ANOVA. A *P*-value <0.05 was considered statistically relevant.

## Results

### Sequence Analysis of the Putative FAD-D glycerol-3-Phosphate Dehydrogenase (gG3PD) of *G. duodenalis* Reveals an Unusual Protein Organization

The GL50803_16125 entry encodes a protein of 1111 amino acids (∼119 kDa) annotated as FAD-dependent oxidoreductase/glycerol-3-phosphate dehydrogenase, that we named gG3PD. A combined search for conserved regions in the gG3PD protein sequence retrieved three principal domains (Supplementary Figure S1A). The region encompassing residues 31–510 contains the multidomain TIGR03377 corresponding to the protein family of the subunit A (GlpA) of anaerobic GlpACB. A second domain, residues 587–910, corresponds to a small NADH binding domain within a larger FAD binding domain (Pyr_redox_2, PF07992) which is common to flavoproteins of the pyridine nucleotide-disulphide oxidoreductases family [e.g., thioredoxin reductases (TRxR), NADH oxidases, and peroxidases]. The last domain (DUF 1667), encompassing residues 1039–1103, was previously found in archaeal and bacterial hypothetical proteins, some of which are annotated as being potential metal-binding proteins. As for other G3PDs, no TM regions could be detected. Due to its unusual domain organization, the full-length or three portions (residues 1–510, 511–950, and 951–111) of the gG3PD protein sequence were used as query against a large non-redundant set of high quality reference genomes obtained from EggNog. Despite an homologous sequence present in the genome of the closely related diplomonad *Spironucleus salmonicida*, only protozoan parasites of the genus *Entamoeba* encode for a single sequence orthologous to the full length gG3PD and also annotated as glycerol-3-phosphate dehydrogenase. One orthologue of the first two portions of the gG3PD was found in the genomes of *Eggerthella* sp., a non-sporulating medically important anaerobic Gram-positive bacillus, and annotated as FAD-dependent oxidoreductase. On the contrary several hundred orthologues of the first portion of gG3PD (residues 1–510), all annotated as glycerol-3-phosphate-dehydrogenases, were found in the genome of human and animal pathogenic bacteria, such as *Treponema* sp. (Spirochaetes) and *Clostridium* sp. (Firmicutes), or to halophilic archaea, e.g., *Halorhabdus utahensis*, (Supplementary Figure S1B). More than 100 orthologues of the second portion (residues 501–950) were found in human and animal pathogenic bacteria, but also in soil and plant bacteria (data not shown). Although several of these proteins were annotated as oxidoreductases it was not possible to define, in terms of substrate or biochemical pathway, any specific enzymatic function. Finally, only 33 orthologues were found respect to the third portion of gG3PD (residues 951–111), which putative function could be that of metal-binding proteins.

### The Intracellular Localization and Stage Expression of gG3PD Protein

**FIGURE 1:**
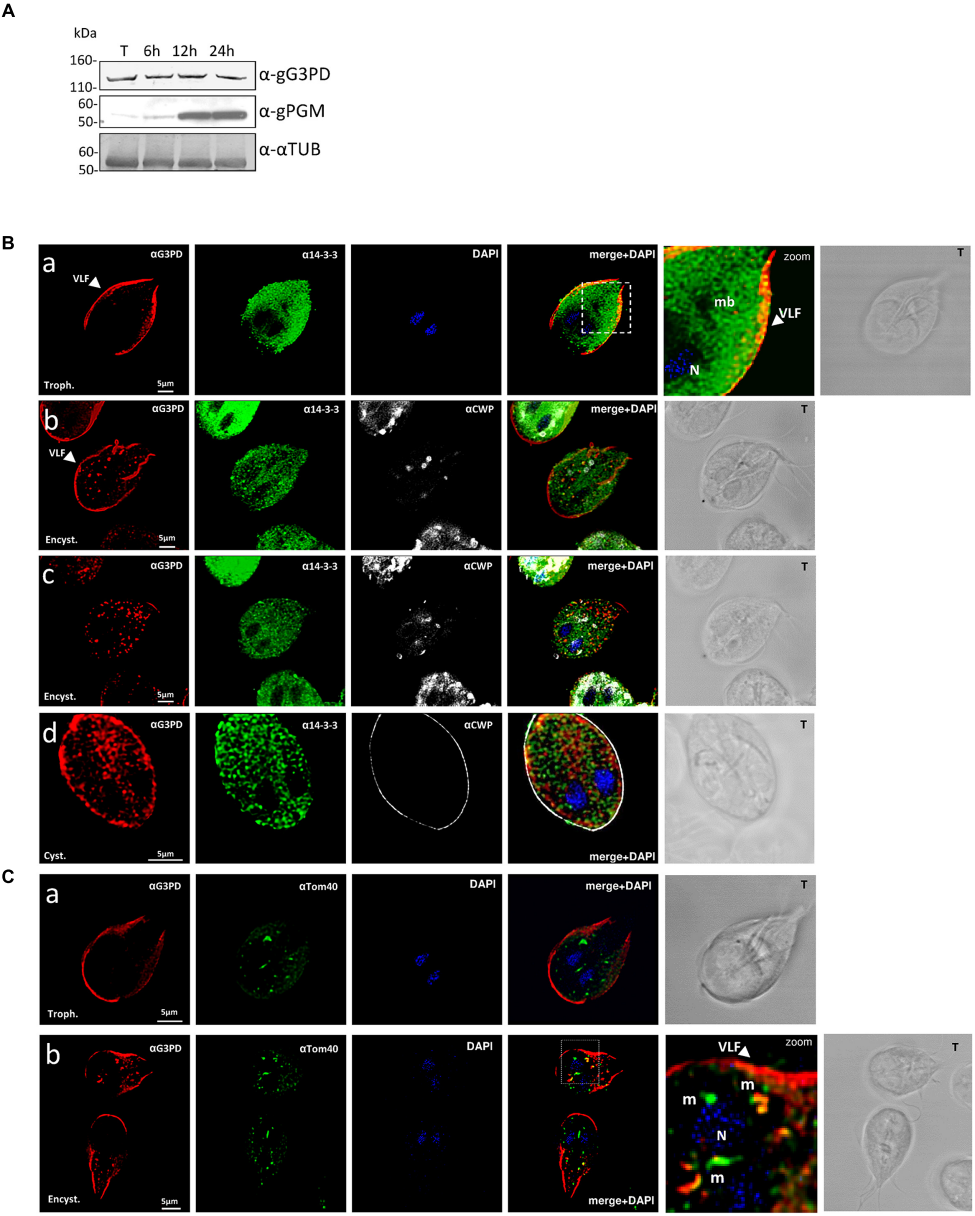
**Expression and localization of the glycerol-3-phosphate dehydrogenase (gG3PD) during the differentiation stages of *Giardia duodenalis*. (A)** Western blot from three independent analysis of Triton lysates (20 μg) from *G. duodenalis* WB-C6 trophozoites (T) and parasites harvested at 6, 12, and 24 h after encystation induction. Immunoblotting was performed with: anti-gG3PD polyclonal serum (α-gG3PD); anti-phosphoacetylglucosamine mutase (α-PGM) (Lopez et al., 2003), to follow the progression of encystation; the anti-α-tubulin (α-α-TUB), as loading control. Molecular size markers (kDa) are reported on the left. The analysis is representative of three independent experiments. **(B)** Confocal laser scanning microscopy (CLSM) observations of fixed and permeabilized *G. duodenalis* WB-C6 parasites at different stages: trophozoite (panel a, Trophozoite), encysting parasite after 12 h of encystation (panel b and c, Encystation) and cyst (panel d, Cyst) stained with the mouse polyclonal serum α-gG3PD (red) and the α-g14-3-3 rabbit polyclonal (green). Cyst wall and encystation specific vesicles (ESVs) were stained with Cy3-conjugated α-CWP mAb (gray). Nuclei (N) were stained with 4′,6-diamidino-2-phenylindole (DAPI; blue). Displayed micrographs correspond to a single z-stack: a and b, ventral stacks; c and d, central stacks encompassing the nuclei. T, transmission light acquisition. Scale bars, 5 μm. Arrows indicate the ventrolateral flanges (VLF). A magnification (zoom) of the indicated area in the merged image is shown. **(C)** CLSM observation as in **(B)**. Parasites were stained with α-gG3PD (red) and α-Tom40 antiserum (green; Dagley et al., 2009). Nuclei (N) were stained with DAPI (blue). A magnification (zoom) of the indicated area in the merged image is shown. Mitosomes are indicated (m). Images in **(B)** and **(C)** are representative of >50 fields analyzed in two independent experiments.

According to the data reported in GiardiaDB, the expression level of the *gg3pd* is downregulated during encystation. To investigate whether the gG3PD protein expression level was similarly regulated we used a mouse polyclonal antibody raised against a full-length HIS-tagged recombinant gG3PD expressed in bacteria. As shown in **Figure 1A**, a band of approximately 120 kDa was immunodecorated with comparable intensity by the anti-gG3PD Ab, both in cellular lysates from trophozoites and from parasites harvested at different time points after encystation induction (at 6, 12, and 24 h). Encystation progress was checked by immunostaining with anti-phosphoacetylglucosamine mutase (gPGN) Ab, a protein induced during cyst formation (Lopez et al., 2003). The anti-gG3PD Ab was also used to study the intracellular localization of the protein by CLSM. In the trophozoite (**Figure 1Ba**) the anti-gG3PD strongly stained the plasma membrane of the parasite ventral surface likely corresponding to the marginal groove and ventrolateral flanges (VLF), fingerlike projections of the plasma membrane involved in parasite attachment to the surface and to the host cell (Sousa et al., 2001). As previously reported, the g14-3-3 showed a spotty broad distribution in the parasite cell body, excluding the nuclei and the median body (smile-like microtubule aggregate in the middle of parasite body). The widespread g14-3-3 staining makes it difficult to claim for a discrete co-localization with the gG3PD, although a slight co-localization signal (pseudo color yellow) was visible at the posterior edge of the trophozoite (**Figure 1Ba**, merge). *G. duodenalis* encystation was monitored by the progressive appearance of specialized structures for the secretion of the cyst wall proteins (CWP), the encysting specific vesicles (ESVs), which were stained, together with the cyst wall, by the anti-CWP mAb. In encysting parasites, in addition to the ventral plasma membrane, the anti-gG3PD Ab (**Figures 1Bb,c**) labeled several structures, similar to aggregates/small vesicles, in the cytoplasm. These structures did not co-localize with the ESVs and no straightforward co-localization with g14-3-3 could be detected (**Figures 1Bb,c**). In cysts, the gG3PD was diffused in the cytoplasm, although a marked aggregation close to the plasma membrane, below the cyst wall was evident (**Figure 1Bd**). A faint co-localization with g14-3-3 (pseudo color yellow) was detected below the cyst wall.

Since the staining with the anti-gG3PD Ab observed in encysting parasites resembled that of mitosomes, additional co-localization experiments were performed using an antibody directed against the mitosomal marker Tom40 (Dagley et al., 2009). As shown in **Figures 1Ca,b**, both in trophozoites and encysting parasites, the anti-Tom40 Ab stained the main central mitosome between the nuclei and several peripheral smaller mitosomes. In trophozoites no co-localization with anti-G3PD could be detected (**Figure 1Ca**), whereas in encysting parasites superimposable signals (mean average 42 ± 6%; **Figure 1Cb**, pseudo color yellow) were mainly evident in the peripheral mitosomes (mean average 27 ± 5%), and to a lesser extent in the central one (mean average 2 ± 0.5%), suggesting a partial re-localization of the protein to mitosomes.

### Interaction between gG3PD and g14-3-3

**FIGURE 2:**
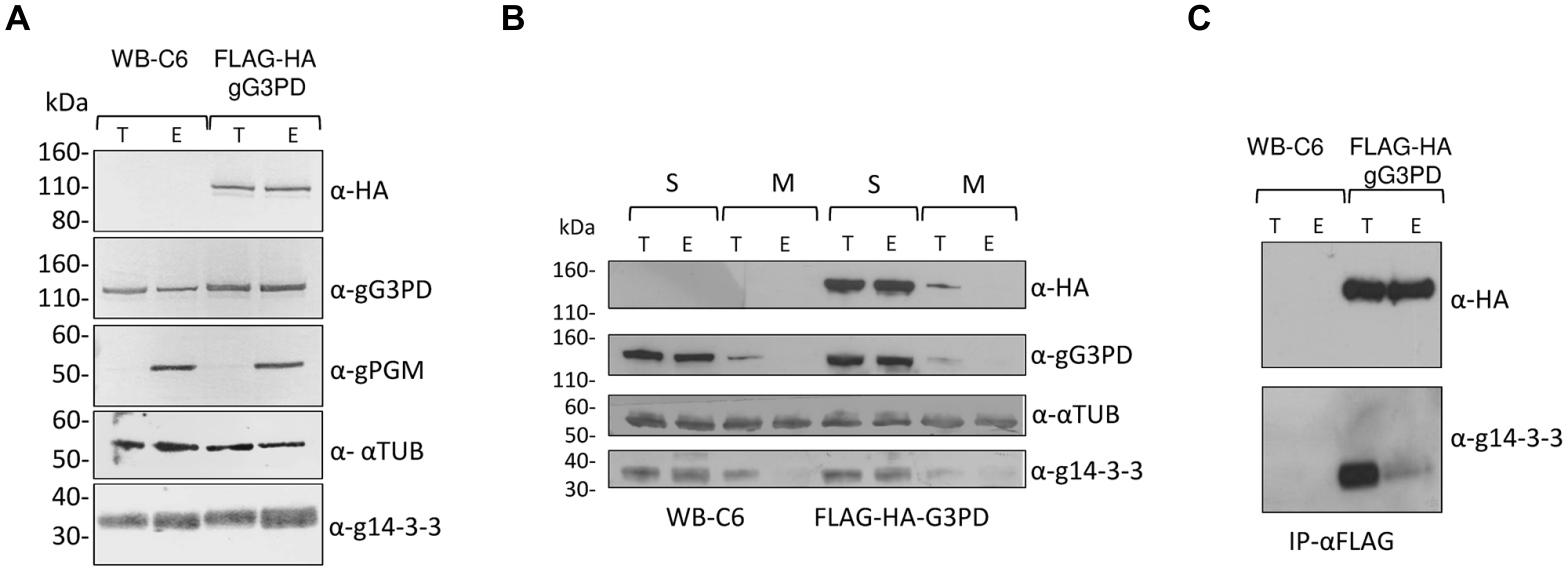
**Expression of the recombinant FLAG-HA-gG3PD in *G. duodenalis* and its interaction with g14-3-3. (A)** Western blot analysis of Triton lysates from trophozoites (T) or parasites after 12 h of encystation (E) obtained from FLAG-HA-gG3PD transfected line or from the control WBC6 strain. Twenty-microgram of protein extracts were loaded in each lane and separated on a 4–12% SDS-PAGE, then transferred on nitrocellulose membrane and finally probed with the indicated antibodies. Equal protein loading was monitored by anti-TUB Ab, whereas encystation induction was confirmed by anti-gPGM Ab. Molecular size markers (kDa) are reported in the left. **(B)** Subcellular distribution of gG3PD. Thirty-microgram of fractionated protein lysate, soluble (S) or octyl β-D-glucopyranoside solubilized membrane proteins (M), from trophozoites (T) or 12 h encysting parasites (E) of FLAG-HA-gG3PD transfected parasites or control WB-C6, were treated as described in **(A)** and probed with the indicated antibodies. Molecular size markers are on the left. **(C)** Co-immunoprecipitation assay of endogenous g14-3-3 with FLAG-HA-gG3PD. An aliquot (1:5) of the FLAG peptide-eluted material from WB-C6 or FLAG-HA-gG3PD transgenic line, deriving from trophozoites (T) or encysting parasite (E), was separated on 4-12% SDS-PAGE and immunoblotted with anti-HA (α-HA) or anti-g14-3-3 polyclonal serum (α-g14-3-3). Molecular size markers are on the left. Immunoblots of **(A–C)** are representative of three independent experiments.

To further characterize the gG3PD, a N-terminally FLAG-HA tagged gG3PD was expressed in *G. duodenalis* under the α*-tubulin* constitutive promoter. The FLAG-HA-gG3PD was expressed at the same level, and comparable to the endogenous protein, both in trophozoites and during the encystation stage (**Figure 2A**) and the intracellular localization largely resembled that of the endogenous gG3PD (Supplementary Figure S2). By following protein fractionation (soluble and membrane fractions), the endogenous and the FLAG-HA-gG3PD were mainly detected in the soluble fraction at both stages (**Figure 2B**). Whereas, a small protein amount could be detected in the membrane fraction only at the trophozoite stage (**Figure 2B**), partially in agreement with immunolocalization observations, suggesting that the gG3PD could form strong interactions with the membrane or with membrane proteins. To better investigate the previously suggested interaction between g14-3-3 and gG3PD (Lalle et al., 2012), immunoprecipitation experiments were performed. As shown by anti-HA immunoblotting (**Figure 2C**, upper), the anti-FLAG mAb immunoprecipitated the FLAG-HA-gG3PD only from transfected parasite extracts. Despite FLAG-HA-gG3PD was comparably immunoprecipitated from both trophozoites and encysting parasites, g14-3-3 was mainly co-immunoprecipitated from trophozoites, whereas only a faint signal was observed in the immunoprecipitate from encysting parasites (**Figure 2C**, lower).

**FIGURE 3:**
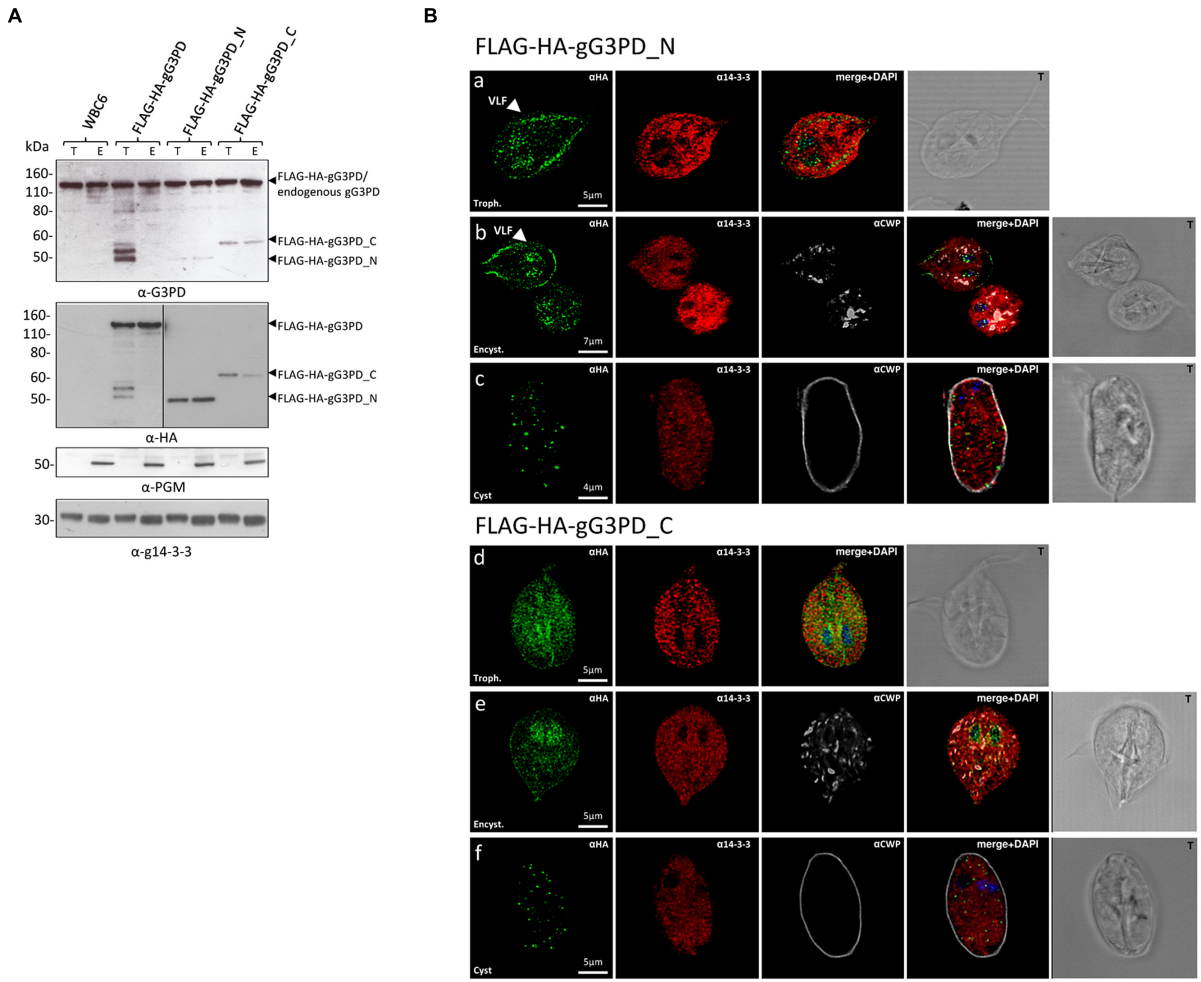
**Expression and intracellular localization of the FLAG-HA-tagged gG3PD deletion mutants in *G. duodenalis* parasites. (A)** Representative western blot analysis of soluble protein lysate (20 μg) from control WB-C6 and transgenic trophozoites (T) or parasites after 12 h of encystation (E), expressing the full-length FLAG-HA-G3PD, or the FLAG-HA-gG3PD_N or _C. Lysates were separated on 4–12% SDS-PAGE and immunoblotted with the indicated antibodies. Arrows on the right indicate the molecular size of the corresponding proteins. Molecular size markers are on the left. The vertical line in the panel corresponding to the α-HA blotting indicates two different times of exposure between the samples of the same gel. The immunoblots are representative of three independent experiments. **(B)** CLSM observations of fixed and permeabilized transgenic *G. duodenalis* parasite expressing the FLAG-HA-gG3PD_N or the FLAG-HA-gG3PD_C at different stages: trophozoite (panels a and d, Trophozoite), encysting parasites after 12 h of encystation (panels b and e, Encystation), and cysts (panels c and f, Cyst). Parasites were stained with mouse α-HA mAb (green) and rabbit polyclonal α-g14-3-3 (red). Cyst wall and encystation specific vesicles (ESVs) were stained by Cy3-conjugated α-CWP mAb (gray). Nuclei were DAPI-stained (blue). Displayed micrographs correspond to a single z-stack. T, transmission light acquisition. Scale bars are reported. Arrows indicate the ventral plasma membrane and VLF. Images are representative of >50 fields analyzed in two independent experiments.

To shed light on the relevance of the two principal domains characterizing the gG3PD protein, two deletion mutants were constructed and independently expressed in *G. duodenalis* as FLAG-HA-tagged proteins. The FLAG-HA-gG3PD_N corresponds to N-terminal half of the protein (Supplementary Figure S1A) and contains the FAD-dependent anaerobic glycerol-3-phosphate dehydrogenase-like domain (residues 2–530); whereas the FLAG-HA-gG3PD_C corresponds to the C-terminal half and contains the FAD-dependent pyridine nucleotide-disulphide oxidoreductase-like domain (residues 524–1111). As shown by immunoblot (**Figure 3A**, upper and middle), the expression of both FLAG-HA-tagged proteins was detected at both stages. In all transfection experiments, the expression of deletion mutants was lower than that of the full-length FLAG-HA-gG3PD.

Intracellular localization of both mutants was performed with anti-HA antibody. CLSM analyses (**Figures 3Ba,b**) showed that, in trophozoites and encysting parasites, the FLAG-HA-gG3PD_N partially localized to the ventral plasma membrane and VLF, like the endogenous gG3PD. Although a spotty cytoplasmic and an intense nuclear/perinuclear staining were evident, in contrast with the endogenous and the full-length FLAG-HA-gG3PD, suggesting that a fraction of the deleted protein could be misfolded and/or mislocalized. In FLAG-HA-gG3PD_C transgenic parasites the protein was completely mislocalized and appeared widespread distributed, also inside nuclei, with the nuclear/perinuclear localization even more evident in encysting parasites (**Figures 3Bd,e**). No mitosome-like staining was observed in encysting parasites for any of the mutants (**Figures 3Bb,e**). Few cysts with a spotty cytoplasmic localization of the protein were observed in both transgenic strains (**Figures 3Bc,f**). These observations likely suggest, that the N-terminal half of the protein contains the structural/sequence determiners necessary to localize the gG3PD at the ventral plasma membrane and VLF. Looking for the domain(s) involved in the interaction with g14-3-3, co-immunoprecipitation with anti-FLAG was also performed using the FLAG-HA deletion mutants. Despite several attempts no conclusive results were obtained (data not shown), likely due to the low expression of these proteins or to the presence of other factors that could affect the interaction (e.g., partial misfolding, incomplete phosphorylation).

### Evaluation of gG3PD Enzymatic Activity

**FIGURE 4:**
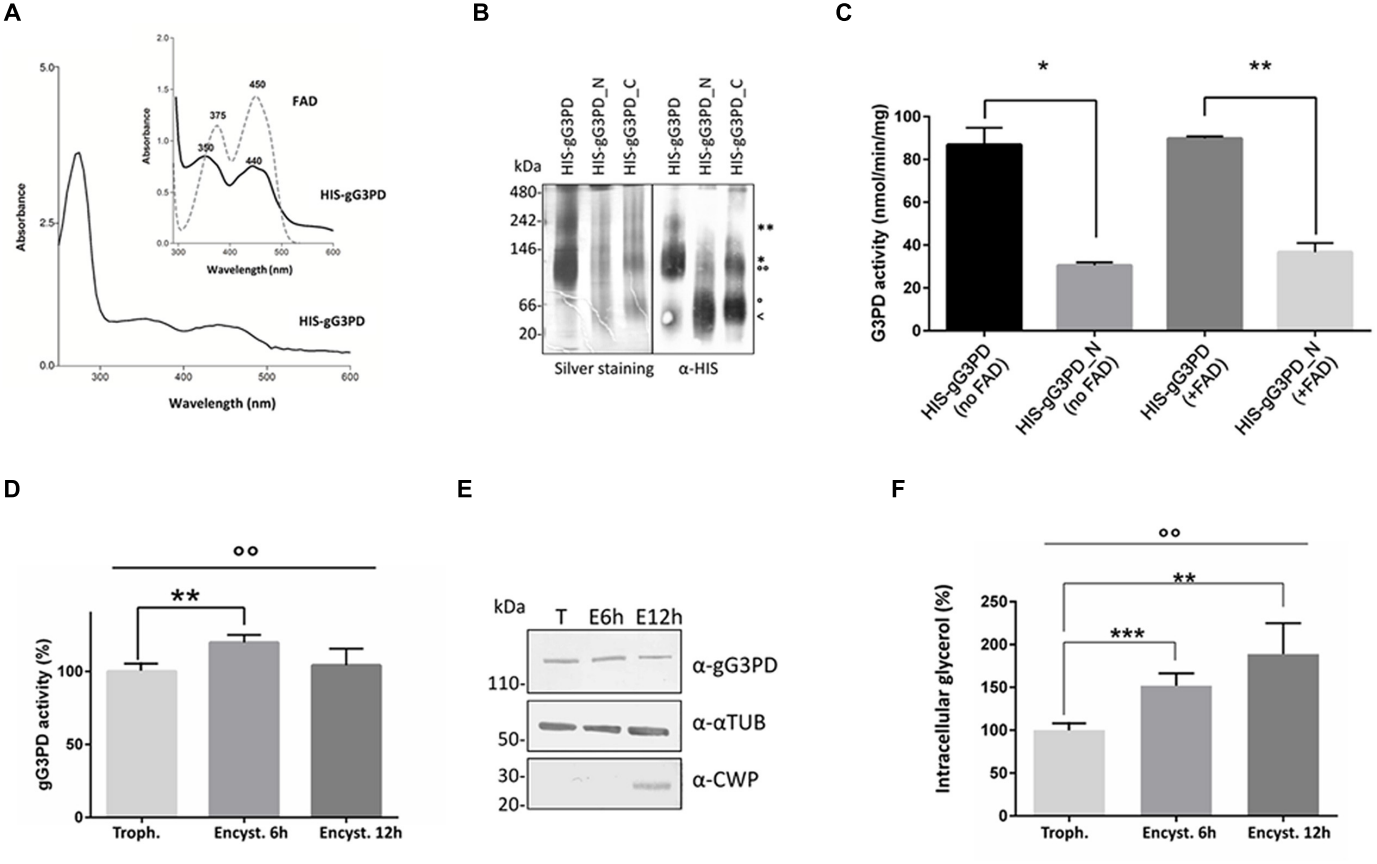
**Evaluation of the gG3PD enzymatic activity. (A)** Spectrophotometric analysis of purified HIS-gG3PD. The UV-visible spectrum of HIS-gG3PD (10 mg/ml) in 67 mM of potassium phosphate buffer, pH 7.5, was recorded at 25°C. The insert shows a magnification of the HIS-gG3PD spectrum (solid line) in comparison with the spectrum of authentic FAD (dotted line) recorded in the same buffer. Peak maxima are reported. Spectra are representative of three independent experiments. **(B)** Assessment of HIS-gG3PD dimerization *in vitro*. Purified recombinant proteins (3 μmol) were separated on 3–12% Blue Native-PAGE and silver-stained or transferred on polyvinylidene difluoride (PVDF) membrane and probed with anti-HIS mAb. Native size markers (kDa) are indicated on the left. Asterisks indicate HIS-gG3PD monomer (^∗^) or dimer (^∗∗^). Empty dots indicate HIS-gG3PD_C monomer (°) or dimer (^°°^). The arrow indicates HIS-gG3PD_N monomer (<). Native-PAGE and immunoblot are representative of three independent experiments. **(C)** The enzymatic activities of both the purified HIS-gG3PD and the deletion mutant HIS-gG3PD_N were measured *in vitro*, by MTT assay, in the presence (+FAD) or absence (no FAD) of 10 μM FAD. The G3PD activity (mean ± SD) from three independent experiments is expressed as nmol of reduced MTT per min per mg of recombinant protein. Statistical analyses were performed using unpaired *t*-test between the full length and the deletion mutant: ^∗^*P* < 0.05 and ^∗∗^*P* < 0.01. **(D)** The FAD-glycerol-3-phosphate dehydrogenase activity was measured in protein extract (100 μg) from *G. duodenalis* trophozoites (Trophozoite) or in parasite after 6 or 12 h from encystation induction (Encystation). The relative enzymatic activity (mean ± SD) from three independent experiments is expressed as the percentage change respect to the value measured in trophozoites. Statistical analyses were performed using unpaired *t*-test [Trophozoite vs. Encystation 6 h (^∗∗^*P* < 0.01) and Trophozoite vs. Encystation 12 h (ns)], and one-way ANOVA (^°°^*P* < 0.001). **(E)** Western blot analysis of protein extracts (20 μg) used to assay the gG3PD enzymatic activity (as described in **D**), extracts were separated on 4–12% SDS-PAGE and immunoblotted with the indicated antibodies. Molecular size markers are indicated on the left. Immunoblot is representative of three independent experiments. **(F)** The intracellular glycerol content was measured in supernatant from *G. duodenalis* trophozoites (Trophozoite) and parasites after 6 or 12 h from encystation induction (Encystation). The relative glycerol amount of three independent experiments (mean ± SD) is expressed as the percentage change respect to the amount detected in trophozoites. Statistical analyses were performed using unpaired *t*-test between Trophozite and Encystation 6 h (^∗∗∗^*P* < 0.001) and Trophozite vs Encystation 12 h (^∗∗^*P* < 0.01). One-way ANOVA confirmed that differences among groups were statistically significant (^°°^*P* < 0.001).

To better define the role of gG3PD during *G. duodenalis* differentiation, we first characterized the protein activity using N-terminally HIS-tagged versions of both full-length gG3PD and deletion mutants heterologously expressed in *E. coli*. All recombinants were expressed as soluble proteins and could be purified (up to 95%) under native conditions (Supplementary Figure S3). The purified full-length HIS-gG3PD, but not the deletion mutants, showed a distinctive yellow color (data not shown), compatible with the presence of a flavin cofactor. Indeed, the UV-visible spectra of the HIS-gG3PD showed two absorbance peaks with a maximum approximately at 350 and 440 nm (**Figure 4A**), very close to those of free flavin (either FAD or FMN or both), supporting that the gG3PD is a flavoprotein. No similar peaks could be observed in the spectra of any deletion mutants (data not shown). Since mG3PD/glpD are homodimeric proteins (Mráček et al., 2013), the dimeric nature of gG3PD was assayed using native PAGE. As shown in **Figure 4B**, the HIS-gG3PD_N was exclusively monomeric (60 kDa); whereas the HIS-gG3PD migrated in the gel both as a 120 kDa monomer and as a 240 kDa dimer, as confirmed by immunoblot assay with anti-HIS. Similarly, the HIS-gG3PD_C migrated as a monomer around 60 kDa, and as a dimer of 120 kDa, thus suggesting that at least one function of the gG3PD C-terminal half is to mediate protein dimerization.

The FAD-glycerol-3-phosphate dehydrogenase activity of the HIS-gG3PD was then assayed by measuring the g3p-dependent reduction of MTT in presence of PMS, which mediates the transfer of reducing equivalent from the enzyme to the terminal dye (Kistler and Lin, 1972). As shown in **Figure 4C**, the HIS-gG3PD displayed a specific activity corresponding to 86.7 ± 5.6 nmol/min/mg in the absence of exogenous FAD, that did not significantly increase (89.8 ± 0.7 nmol/min/mg) even when 10 μM FAD was added. Moreover, no activity could be observed either in the absence of g3p or PMS (data not shown), thus proving that g3p is indeed the substrate of the enzyme and that an electron carrier molecule, such as PMS, is required to reduce MTT. This is in agreement with the role of quinone described for other FAD-dependent G3PDs (Unden and Bongaerts, 1997; Mráček et al., 2013). Intriguingly (**Figure 4C**), the HIS-gG3PD_N, containing the GlpA-like domain, also displayed a basal enzymatic activity (30.5 ± 1.0 nmol/min/mg), 2.8-fold lower in comparison with the full length protein, and it showed an increase of 17% in the activity (36.7 ± 3.0 nmol/min/mg) after stimulation with exogenous FAD. On the contrary, the HIS-gG3PD_C did not show any enzymatic activity in any condition (data not shown). These results support the homology data and confirm that the glycerol-3-phospate dehydrogenase activity is localized to the N-terminal half of the gG3PD.

To study the possible correlation between encystation-dependent intracellular re-localization of gG3PD and its function, the enzyme activity was measured in trophozoites and encysting parasites extracts. Indeed, compared to trophozoites, the gG3PD activity showed a 20% increase at 6 h of encystation (**Figure 4D**), then it was reduced nearly down to the trophozoite level after 12 h of encystation. No variation in the expression level of the gG3PD protein could be observed at any time points (**Figure 4E**). The encystation process in *G. duodenalis* has been proposed as a primitive response to cellular stress (Argüello-Garciá et al., 2009). In unicellular organisms, as response to stress signals, the intracellular level of glycerol increases, as consequence of a massive conversion of DAPH to g3p, due to the greater activity of the NADPH-dependent G3PDs and in some cases also to that of FAD-dependent G3PDs (Yang et al., 2007). To test this hypothesis we measured the intracellular content of glycerol during encystation. The amount of glycerol increased up to 60% already at 6 h post-encystation induction (**Figure 4F**) and then of an additional 15% in 12 h encysting parasites, thus suggesting a possible relationship between the enhanced gG3PD activity and the glycerol accumulation occurring during parasite differentiation.

### *In Vivo* Effects of NBDHEX on *G. duodenalis* Survival and gG3PD Activity

**FIGURE 5:**
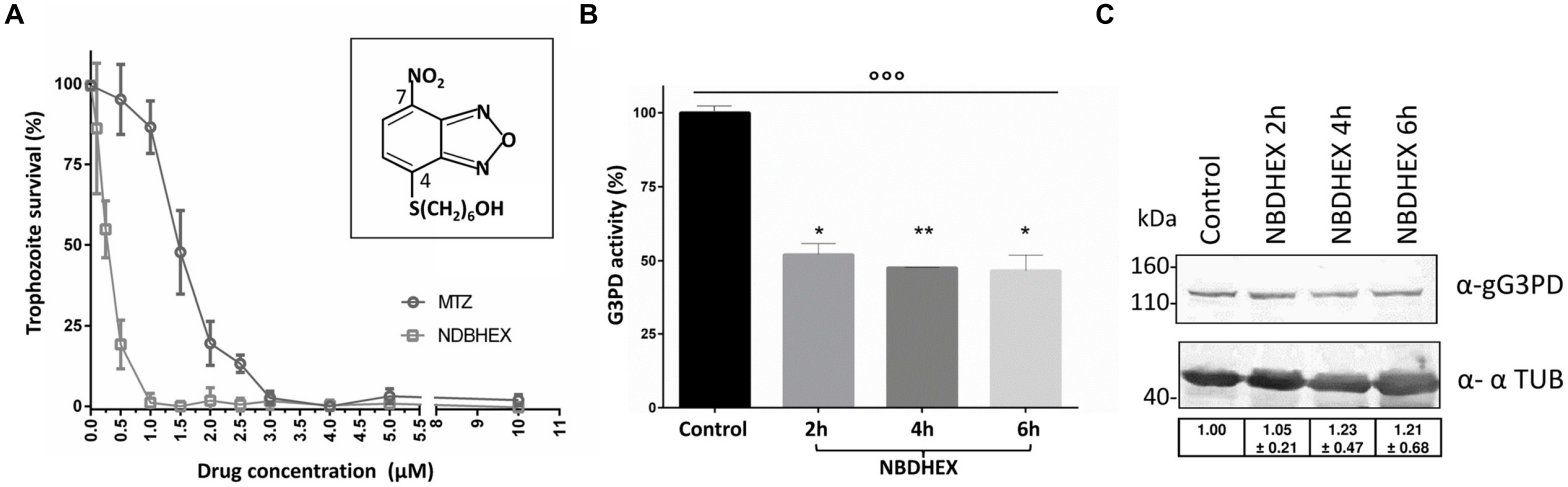
**Evaluation of NBDHEX effects on *G. duodenalis* growth and gG3PD enzymatic activity. (A)** Survival of *G. duodenalis* WBC6 trophozoites was determined by methylene blue colorimetric assay after 48 h of treatment with different concentrations, ranging from 0.05 to 10 μM, of NBDHEX (empty dots) or metronidazole (MTZ, empty squares) in microaerophilic growth conditions. Data (mean percentage ± SD) represent three independent experiments, each done in triplicate. The structure of the NBDHEX compound is reported in the insert. **(B)** The FAD-glycerol-3-phosphate dehydrogenase activity was measured in protein extract (100 μg) from *G. duodenalis* trophozoites treated for the indicated times with 50 μM NBDHEX or for 6 h with ethanol (Control). The enzymatic activity of three independent experiments (mean ± SD) is expressed as the percentage change respect to the control. Unpaired *t*-test was performed between control and each time point (^∗^*P* < 0.05 and ^∗∗^*P* < 0.01). One-way ANOVA indicated statistically significant differences among all stages (^°°∘^*P* < 0.0001). **(C)** Twenty-microgram of protein extracts derived from trophozoites treated as described in **(B)**, were separated on 4–12% SDS-PAGE and immunoblotted. A representative western blot analysis is shown and the antibodies indicated. Molecular size markers are reported in the left. Table in the bottom reports the densitometric analysis of three independent experiments (mean ± SD). Statistical analyses using *t*-test were not significant.

Recently, a 7-nitro benzoxadiazole (NBD) derivative has been shown to impair the growth of PC-3 adenocarcinoma cell line by a significant inhibition of the glycerol-3-phosphate oxidoreductase activity of mG3PD (Singh, 2014). We wondered whether this class of molecules could be effective against *G. duodenalis* trophozoites and the gG3PD. We selected the NBDHEX, a promising antitumoral drug acting as a suicide inhibitor of human glutathione S-transferases (GSTs), that also shows a good tolerance in mouse models (Ricci et al., 2005; Turella et al., 2005; Pellizzari Tregno et al., 2009; Tentori et al., 2011). NBDHEX cytotoxicity against *G. duodenalis* trophozoites was evaluated *in vitro* after 48 h of treatment in microaerophilic growth conditions, using a colorimetric assay. As shown (**Figure 5A**), NBDHEX was effective against the parasite (IC_50_: 0.3 ± 0.1 μM) at a lower concentration (5.6-fold) than that of the reference drug MTZ (IC_50_: 1.5 ± 0.1 μM). Moreover, under anaerobic growth conditions NBDHEX was also more effective than MTZ (IC_50_: 0.6 ± 0.4 μM for NBDHEX and IC_50_: 1.9 ± 0.2 μM for MTZ; data not shown). To maximize in a short time period the possible NBDHEX effects, taking into account the parasite intracellular environment, trophozoites were treated *in vivo* with 50 μM NBDHEX and then the gG3PD enzymatic activity was assayed in parasite extracts. A 50% decrease of the gG3PD activity was already evident after 2 h of treatment (**Figure 5B**, *P* < 0.01), and a further 5% decrease was achieved after 4 and 6 h. This suggests that *in vivo* NBDHEX treatment reduces the gG3PD activity, without inducing any statistically relevant variation of the protein expression level (**Figure 5C**).

### Interaction of NBDHEX with gG3PD

**FIGURE 6:**
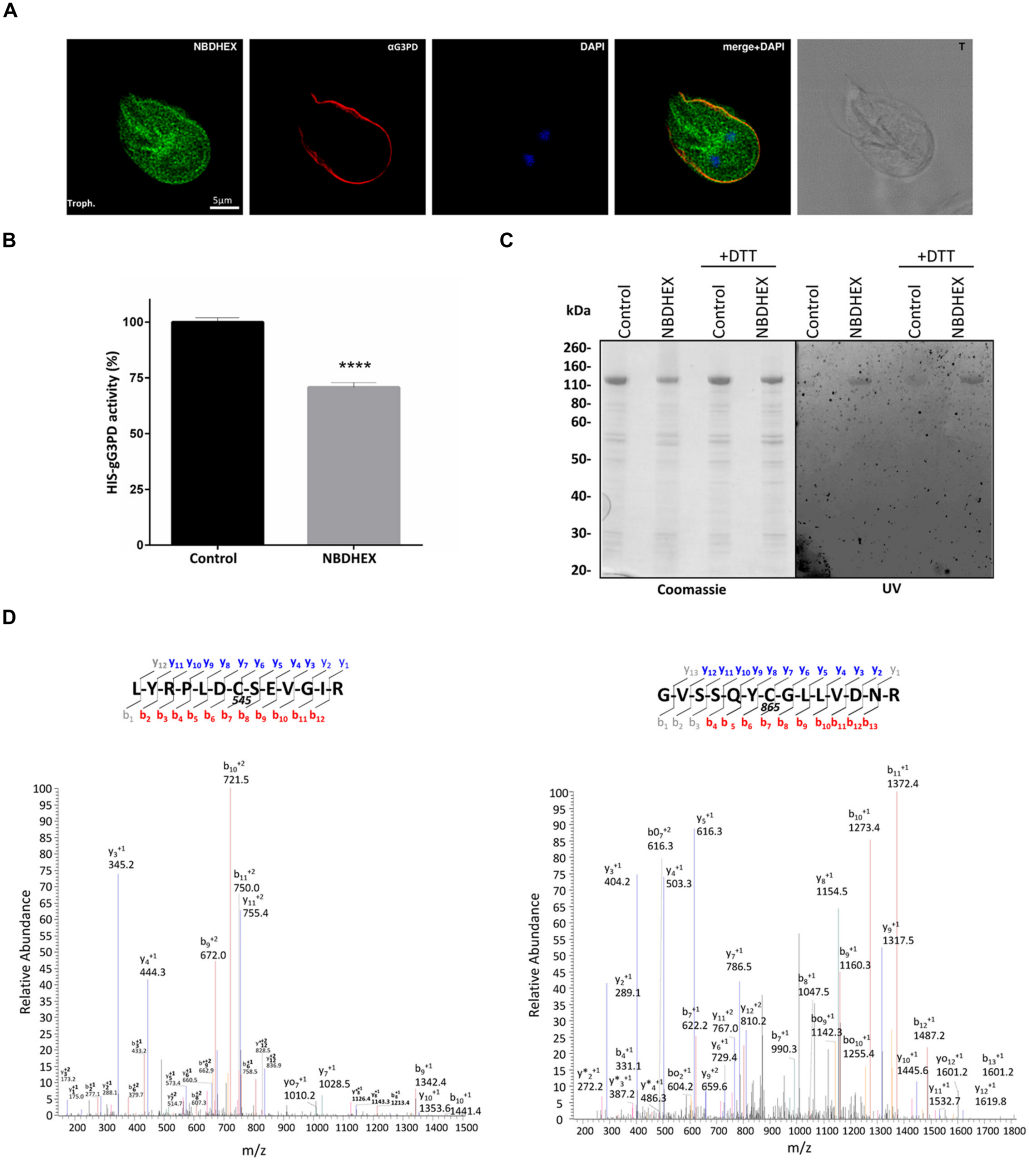
**Interaction of NBDHEX with gG3PD. (A)** CLSM analysis of fixed and permeabilized *G. duodenalis* WBC6 trophozoites after 2 h incubation with 50 μM NBDHEX. NBDHEX (green) was directly visualized using the laser light at 488 nm ex. Parasites were stained with rabbit polyclonal anti-g14-3-3 and AlexaFluor 647-conjugated anti-rabbit (red). Nuclei were stained with DAPI (blue). Displayed micrographs correspond to a single z-stack. T, transmission light acquisition. Scale bar is reported. Images are representative of >50 fields analyzed in two independent experiments. **(B)** Histograms represent the FAD-glycerol-3-phosphate dehydrogenase activity (mean percentage ± SD) of recombinant HIS-gG3PD (16 pmol) purified from HIS-gG3PD-overproducing *E. coli* after 2 h incubation with 50 μM NBDHEX (NBDHEX) or ethanol (Control). The relative change in the enzymatic activity is given as percentage in relation to the control. Statistical analyses were performed using unpaired *t*-test (^∗∗∗∗^*P* < 0.0001) on three independent experiments. **(C)** SDS-PAGE (4–12%) of 2 μg of purified NBDHEX-treated (NBDHEX) or ethanol-treated (Control) HIS-gG3PD, as described in **(B)**, under reducing (+DTT) or not reducing condition. On the left, gel stained with Coomassie blue; on the right, the same gel photographed under UV light prior to staining. Molecular size markers are indicated on the left. **(D)** MS/MS spectra matching the gG3PD peptides (residues 539–551, left, and residues 859–872, right) carrying a mass increase of 265 Da on the cysteine residues C545 and C865, respectively. The mass shift is compatible with an NBDHEX-derived adduct in which the nitro group was completely reduced to an amine. Data shown in **(C)** and **(D)** are representative of three independent experiments.

**FIGURE 7:**
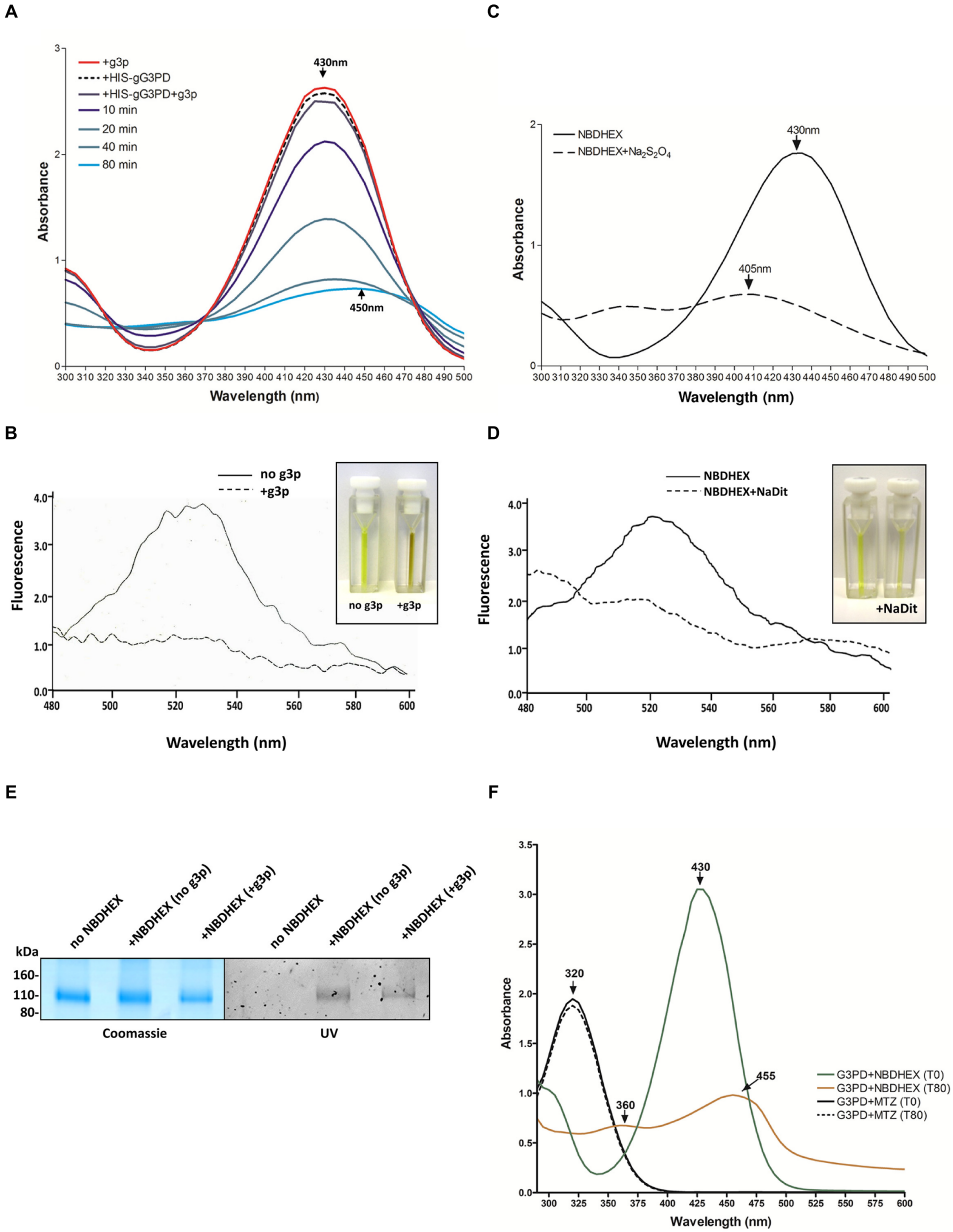
**NBDHEX is substrate of HIS-gG3PD. (A)** Spectrophotometric analysis of the reaction between NBDHEX and gG3PD. The UV-visible spectrum of NBDHEX (100 μM) in 67 mM potassium phosphate buffer (pH 7.5) was recorded at 25°C either in the presence of 17 mM g3p (red line, +g3p) or 80 nM HIS-gG3PD (dashed black line, +HIS-gG3PD). The NBDHEX (100 μM) UV-visible spectrum was also recorded at different time points (0, 10, 20, 40, and 80 min) in the presence of both 17 mM g3p and 80 nM HIS-gG3PD (+HIS-gG3PD+g3p). Arrows indicate the wavelength (nm) of the maximal absorption. **(B)** Fluorescence spectra of 100 μM NBDHEX (excitation at 430 nm) after 80 min of incubation with 80 nM HIS-gG3PD, either in the presence (solid line) or absence (dashed line) of 17 mM g3p. Fluorimetric analyses were recorded at 25°C in 67 mM potassium phosphate buffer (pH 7.5). NBDHEX fluorescence emission has a maximum peak wavelength at 525 nm. Fluorescence intensity (*y* axis) is arbitrary. The change in color of the two reactions is shown in the insert. **(C)** The UV-visible spectrum of NBDHEX (100 μM) in 67 mM potassium phosphate buffer (pH 7.5) was recorded at 25°C before (solid line) and after (dashed line) treatment with sodium dithionite (Na_2_S_2_O_4_). The wavelengths (nm) of the maximal absorption are indicated with arrows. **(D)** Fluorescence spectra of 100 μM NBDHEX (excitation at 430 nm) before (solid line) and after Na_2_S_2_O_4_ (dashed line). The slight change in color of the two conditions is shown in the insert. Spectra and images from **(A–D)** are representative of three independent experiments. **(E)** SDS-PAGE (4–12%) of 2 μg of HIS-gG3PD after 80 min of incubation at 25°C with NBDHEX (+NBDHEX) in the presence or not of g3p, or with ethanol (no NBDHEX). Gel was photographed under UV light (UV, right) before staining with Coomassie blue (Coomassie, left). Molecular size markers are reported in the left. Images are representative of three independent experiments. **(F)** Comparison of the UV-visible spectra of NBDHEX (green and orange line) or MTZ (solid and dashed line) incubated with HIS-gG3PD and g3p (G3PD) at time 0 min and after 80 min of incubation at 25°C (T0 and T80, respectively). Arrows indicate the wavelengths (nm) of the maximal absorption. Spectra are representative of three independent experiments.

To ascertain if the effects on the gG3PD activity were due to a direct NBDHEX binding to the enzyme, co-localization experiments were performed by CLSM (**Figure 6A**), taking advantage of the fluorescent properties of the compound (maximum emission peak at 525 nm; Ricci et al., 2005). Only trophozoites treated with NBDHEX showed a spotted faint fluorescence in the cytoplasm. A more intense signal was observed around the nuclei, in the areas corresponding to the median body, in the intracellular portion of ventral flagella and, intriguingly, at the ventral plasma membrane. As shown, co-localization signal (pseudo color yellow) between gG3PD and NBDHEX was evident only at plasma membrane, without any relevant alteration in the intracellular localization of gG3PD (for comparison see **Figures 1B,Ca**). To further study the interaction between NBDHEX and gG3PD, *E. coli* expressing the HIS-gG3PD was incubated with NBDHEX, then the recombinant protein was purified and, finally, the enzymatic activity was assayed. Similar to the *in vivo* treatment of *G. duodenalis* with NBDHEX, incubation of bacteria with the compound resulted in a recombinant HIS-gG3PD having a 25% reduced enzymatic activity (**Figure 6B**). Next, we wondered whether NBDHEX could bind HIS-gG3PD. We take advantage of the property of NBD derivatives covalently bound to a protein to be visualized by fluorescence under ultraviolet (UV) light after SDS-PAGE (Bragg and Hou, 1999). Indeed, HIS-gG3PD, purified from NBDHEX treated bacteria, was fluorescent under UV-light (**Figure 6C**, right), even in the presence of a reducing agent (such as DTT). On the contrary, no fluorescence was associated with the HIS-gG3PD purified from untreated bacteria. These results suggest that NBDHEX strongly binds HIS-gG3PD. To confirm this hypothesis, purified HIS-gG3PD from bacteria, treated or not with NBDHEX, was subjected to mass spectrometry analysis. MS/MS spectra were acquired matching two HIS-gG3PD peptides carrying NBDHEX derived adducts on cysteine residues: the detected mass shift was compatible with an NBDHEX form in which the nitro group was reduced to amine (**Figure 6D**). No adduct with the intact NBDHEX moiety could be found, probably because it was unstable or not detectable under the applied experimental conditions. The presence of nitro-reduced NBDHEX adducts was in favor of an electrochemical reduction of the drug, likely by gG3PD. Therefore, NBDHEX properties were evaluated by spectrophotometric and fluorimetric analyses after *in vitro* incubation with HIS-gG3PD. The UV-visible spectra of NBDHEX, incubated either with g3p or with HIS-gG3PD, showed the typical peak centered around 430 nm (**Figure 7A**), even after 80 min of incubation (data not shown). Incubation in the presence of both HIS-gG3PD and g3p led to the progressive decrease of the 430 nm absorption peak and to the appearance of a less intense new one around 450–455 nm (**Figure 7A**). The reaction was also associated with a change in color, from bright yellow to brown (**Figure 7B**, insert) and the disappearance of the 525 nm emission peak of NBDHEX in the fluorescence spectra (**Figure 7B**). Since the amount of NBDHEX was in large excess compared to HIS-gG3PD, the observed spectral alterations were not imputable to binding of NBDHEX to the enzyme, but they argued on NBDHEX modification by the glycerol-3-phosphate dehydrogenase activity of HIS-gG3PD. Such modifications could be ascribable to a partial or complete reduction of the nitro group to hydroxylamine or amine. Chemical reduction of the NBDHEX nitro group to amine by sodium dithionite, proved by mass spectrometry analysis (Supplementary Figure S4), resulted in the decrease of the NBDHEX absorption peak at 430 nm, although with the appearance of a new peak at 405 nm (**Figure 7C**), and loss of fluorescence too (**Figure 7D**). The observed differences between UV-visible spectra of HIS-gG3PD and sodium dithionite treated NBDHEX suggest that the enzymatic activity produced an optical species, probably compatible with an incomplete reduced form of NBDHEX, carrying an hydroxylamine instead of an amine moiety. On the other side, looking to the NBDHEX-treated HIS-gG3PD, both in the presence or absence of g3p, the enzyme resulted fluorescent under UV light after SDS-PAGE (**Figure 7E**). This suggests that protein adducts with the unreduced NBDHEX occurred despite, enzyme activation. Products from *in vitro* reactions between HIS-gG3PD and NBDHEX were also analyzed by mass spectrometry. In the sample incubated in the presence of g3p, some HIS-gG3PD cysteine residues were modified by NBDHEX and MS/MS spectra analysis allowed to detect mass shifts compatible with NBDHEX adducts carrying the NO_2_ moiety completely reduced to NH_2_, but also the semi-reduced hydroxylamine group (Supplementary Figure S5). These adducts could not be detected when g3p was omitted.

Since toxicity of the nitrocompound MTZ over *G. duodenalis* has been related to its reduction to nitrosoimidazole or hydroxylamine intermediate by flavin enzymes (Leitsch et al., 2007, 2009, 2011), we asked whether the gG3PD could be able to nitroreduce MTZ. We then monitored variation in the MTZ absorbance at 320 nm (Leitsch et al., 2011). As shown, no decrease in MTZ absorbance occurred even after 80 min of incubation in the presence of HIS-gG3PD and g3p (**Figure 7F**), thus suggesting that in our experimental conditions MTZ is not nitroreduced by gG3PD.

## Discussion

In this work we have characterized, for the first time, the FAD-dependent glycerol-3-phosphate dehydrogenase of *G. duodenalis* both at molecular and functional level, and we have shown that the antitumoral 7-NBD derivative NBDHEX displays a remarkable antigiardial activity, targets the gG3PD and, when *in vivo* administered to *G. duodenalis*, induces gG3PD activity reduction.

The full length gG3PD has no orthologues in other organisms, including humans, except for the diplomonad *S. salmonicida* and the amebidae *Entamoeba* sp. The presence in the gram-positive anaerobic actinobacteria *Eggerthella* sp. of an orthologue of the first 950 amino acids suggests that a first gene fusion event between a GlpA-like gG3PD and a pyridine nucleotide-disulphide oxidoreductase occurred in this prokaryotic lineage. A lateral gene transfer (LGT) event could account for the presence of this multi-domain protein both in *G. duodenalis* and *Entamoeba* sp., that share with *Eggerthella* sp. the mammalian intestine as ecological niche. LGT events have been already well documented for genes of the anaerobic metabolism in eukaryotic protozoan parasites such as *Entamoeba histolytica*, *Trichomonas vaginalis*, and *G. duodenalis* (as well as in *S. salmonicida*; Andersson et al., 2007; Morrison et al., 2007; Alsmark et al., 2009).

We demonstrate that the gG3PD is an active flavoenzyme able to use g3p as source of reducing equivalents. In agreement with the domain topology and homology with bacterial GlpA subunit, the enzymatic activity, as proven by deletion mutants, resides in the N-terminal half of the protein. It is worth noting that the modest stimulatory effect exerted by additional FAD on the enzymatic activity of both the gG3PD and the gG3PD_N, could be explained by different reasons (i) the full length gG3PD is fully complexed with FAD; and/or (ii) the deletion mutant lacks the ability to properly bind FAD; and/or (iii) the enzyme needs FMN in addition to FAD. Indeed, the maximal activity of *E. coli* trimeric GlpACB *in vitro* requires both FMN and FAD (Cole et al., 1988), whereas glpD binds only FAD (Yeh et al., 2008). Furthermore, we show that gG3PD is able to dimerize and that the C-terminal half is required for the dimer formation. The dimerization properties of this gG3PD region, absent in bacterial GlpA, could be ascribable to its homology with members of the FAD-dependent pyridine nucleotide-disulphide oxidoreductases, a family of generally dimeric proteins (Argyrou and Blanchard, 2004).

Localization of the gG3PD to the ventral plasma membrane, likely in correspondence to the VLF, and the detection of the enzymatic activity in parasite extracts are in favor of the existence of functional glycerol biosynthetic pathway and of a role of the protein in *G. duodenalis* energy metabolism. Similar to bacteria (Unden and Bongaerts, 1997), metabolization of g3p by *G. duodenalis* could occur at plasma membrane via gG3PD thus providing an extra source of DHAP for glycolysis and reducing equivalents to putative ETCs. Experimental evidences indicate that *G. duodenalis* can proliferate *in vitro* even in absence of glucose, its main energy source (Adam, 2001), either in the presence of serum (Schofield et al., 1991) or of a defined mixture of bile salts, phosphatidylcholine (PC) and cholesterol (Gillin et al., 1986; Gault et al., 1987). Incorporation of free glycerol and g3p in trophozoites has been reported to be negligible (Jarroll et al., 1981). Nevertheless, mammalian bile and intestinal epithelium mucus are rich of PC, that is efficiently taken up by *G. duodenalis* trophozoites (Stevens et al., 1997). PC, in turns, can potentially be converted to g3p by the combined activity of a phospholipase PLB and a putative GPC-PDE, both encoded by *G. duodenalis* genome (Morrison et al., 2007; Yichoy et al., 2011).

We show that the gG3PD activity increases early at encystation, without up-regulation of protein expression. In this phase of encystation, energy metabolism and galactosamine synthesis require extra carbon sources as consequence of the highly reduced glucose uptake and decrease in oxygen consumption (Paget et al., 1998). We may speculate that gG3PD could provide extra carbon to glycolysis, via an improved metabolization of g3p to DHAP. In parallel, we also observed an increase in intracellular glycerol levels. In response to stress conditions, including hyperosmotic stress, several microorganisms synthesized glycerol as stress protector via an increased DHAP to g3p conversion mediated by the NAD(P)H-dependent G3PD (Albertyn et al., 1994; Yang et al., 2007; Chen et al., 2012; Suescún-Bolívar and Thomé, 2015). The increase in the bile salt, together with a slight alkaline pH, is a key encystation signal eventually perceived by *G. duodenalis* as hyperosmotic stress. It is worth to note that, in *E. histolytica*, intracellular accumulation of both g3p and glycerol occurs in response to oxidative stress despite only the gG3PD orthologue is present and no NAD(P)H-dependent G3PD activity has been detected (Husain et al., 2012). The unusual domain architecture of FAD-dependent G3PDs from both *G. duodenalis* and *E. histolytica* may explained for a bi-directional activity of this enzymes A further characterization of the gG3PD and a detailed metabolomics analysis of the trophozoite and encystation stage of *G. duodenalis* will be necessary to unravel any metabolic re-arrangement occurring during the parasite differentiation and its link with gG3PD activity.

The subcellular localization of gG3PD requires also other considerations. The presence, only in trophozoites but not in encysting parasites, of a fraction of the enzyme in the membrane preparation is in agreement with the immunolocalization data and suggests that gG3PD can associate with membranes in a stage dependent-manner. The structural/sequence determinant(s) responsible for the localization of the protein may reside in the N-terminal half of gG3PD, as supported by the partial localization of the gG3PD_N deletion mutant to the ventrolateral side of the trophozoite. In addition, we confirm that gG3PD and g14-3-3 co-precipitate (this work; Lalle et al., 2012) mainly at the trophozoite stage implying a role of the g14-3-3 in gG3PD localization to ventral plasma membrane. The presence of several putative mode-1 and mode-2 binding sites along the sequence of gG3PD (Supplementary Figure S1; Lalle et al., 2012) can allow for a direct interaction between the two proteins. Nevertheless, we cannot exclude that the interaction between gG3PD and g14-3-3 is indirect and that both proteins are present in a multiprotein complex, and further studies with different deletion and point mutants will clarify this issue. However, beyond the exact interaction mechanism, a clear relationship between g14-3-3 and gG3PD localization and, possibly, its enzymatic activity, is indicated by the strongly reduced co-precipitation of g14-3-3 with gG3PD during encystation and the partial re-localization of gG3PD to mitosomes.

The re-localization of gG3PD to mitosomes, despite the absence of any predictable mitochondrial targeting sequence or cleavage site in the N-terminus, has been proved at least for another *G. duodenalis* protein, namely GiiscS (Dolezal et al., 2005). In our case, the lost of mitosomal localization in both gG3PD deletion mutants suggests that targeting to these organelles at least requires the full-length protein. Although *G. duodenalis* mitosomes clearly miss the TCA cycle and the typical ATP-generating mitochondrial respiratory ETC (Han and Collins, 2012), the encystation-dependent localization of gG3PD to mitosomes could be a reminiscence of mitochondrial function of mG3PDs, where they participate in the g3p shuttle and supply electrons to the mitochondria ETC. A homolog of FAD-dependent mG3PD has been identified also in the mitosomes of the microsporidia *Antonospora locustae*, a group of spore-forming fungus-related intracellular parasites (Dolgikh et al., 2011). The protein is expressed in the spore and localizes in the mitosomes, where it channels reducing equivalent to the so called alternative oxidase (Dolgikh et al., 2011). Similarly, the presence of alternative ETCs in *G. duodenalis*, as in the anaerobic bacteria respiration, cannot be excluded (Unden and Bongaerts, 1997; Han and Collins, 2012). GiOR-1, a di-flavoprotein able to reduce the gCYTb5-IV and having ferredoxin-independent and NADPH-dependent reductase activity, has been localized to the *G. duodenalis* mitosome, suggesting that a NAD(P)-dependent ETC exists in this organelle (Jedelský et al., 2011; Pyrih et al., 2014). Similarly, the presence of a pyridine nucleotide-disulphide oxidoreductase domain and of a putative metal binding domain in gG3PD, both present in the flavoprotein reductase family (Argyrou and Blanchard, 2004), may imply that the protein possesses a combined dehydrogenase and reductase activity, although the final acceptor, if any, is still to be identified. Our results disclose a new perspective on the role of mitosome in *G. duodenalis* suggesting that mitosomal components and functions can change during the parasite life cycle. Indeed, gG3PD was not detected in the proteome of mitosomes isolated from trophozoites (Jedelský et al., 2011).

Looking for compounds affecting the gG3PD activity, we demonstrate that the 7-NBD derivative NBDHEX, a patented anti-tumor drug (Ricci et al., 2005; Turella et al., 2005), exerts cytotoxic activity toward *G. duodenalis* trophozoites and, when administered *in vivo*, hampers the g3p dehydrogenase activity of gG3PD. The IC_50_ of NBDHEX is twofold to fivefold lower than MTZ, proving that the compound is more effective in killing *G. duodenalis* than the reference drug, even in microaerobic conditions. This indicates that the presence of O_2_ does not produce opposite effects on NBDHEX, as on the contrary occurs for re-oxidation of MTZ nitroradical anions. *G. duodenalis* is a microaerophilic parasite that lives in the fairly aerobic (up to 60 μM O_2_) environment of the upper intestine, so that a drug potentially effective also in presence of O_2_ is more attractive for therapeutic use. We show that NBDHEX binds to gG3PD, when administered both *in vivo* and *in vitro*. NBDHEX is an excellent electrophile that can easily form σ-complexes, as occurs between glutathione and the C-4 position of the drug in the G-site of GST, leading to the inhibition of the detoxifying and anti-apoptotic activity of human GSTP1-1 (Ricci et al., 2005; Federici et al., 2009). Since no gene coding for GST is present in *G. duodenalis* (Morrison et al., 2007), we can *bona fide* exclude that antigiardial activity of NBDHEX may be due to any GST inhibition, although a low level of GSH has been detected (Krauth-Siegel and Leroux, 2012). The formation of σ-complexes likely occurs between NBDHEX and gG3PD, as suggested by in gel fluorescence of gG3PD after NBDHEX treatment, but, unfortunately, our attempts to identify these complexes by mass spectrometry were unsuccessful. Furthermore, we demonstrate the occurrence of NBDHEX covalent adducts at several gG3PD cysteine residues, with the 7-nitro group of the drug being reduced either completely to amine or partially to hydroxylamine. Different NBDHEX modified cysteine residues of His-gG3PD were found in different experimental conditions, i.e., NBDHEX treatment *in E. coli* or *in vitro*, likely as the result of a differential accessibility of the drug to such residues. Our *in vitro* assays and MS data indicate that the nitro reduced NBDHEX adducts derive from the gG3PD-mediated electrochemical reduction of the drug, possibly via the formation of highly reactive intermediates, such as nitroso or hydroxylamine radicals. It is well known that during the FAD-mG3PD-mediated oxidation of the 2-hydroxy group of g3p, to form DHAP, two electrons are transferred to FAD and then to the quinone pool (Mráček et al., 2013). Similarly, gG3PD activity can generate two electrons able to reduce the nitro group to a hydroxylamine intermediate. Enzymatic reduction has also been proposed as a mechanism for the activation of nitrocompounds, i.e., MTZ, leading to protein covalent adducts formation and cytotoxicity in several microaerophilic protozoan parasites. In particular, the flavoprotein TRxR can nitroreduce MTZ, by a process involving its flavin cofactor, thus forming covalent adducts with the drug (Leitsch et al., 2007, 2009, 2011). Remarkably, although gG3PD displays nitroreductase activity toward NBDHEX, no nitroreduction of MTZ could be observed in our experimental conditions. Nitroimidazoles and other nitrocompounds seem to be metabolized in *G. duodenalis* in a different manner, also in comparison to *E. histolytica* and *T. vaginalis*, and protein adducts can be formed only with some of these drugs (Leitsch et al., 2012). Indeed, gG3PD was not identified among the proteins forming adducts with MTZ or tinidazole neither in *G. duodenalis* nor in *E. histolytica* (Leitsch et al., 2007, 2012). In particular, these differences in nitrocompound metabolization have been related to the adaptation of *G. duodenalis* to rather high concentrations of O_2_ in the upper small intestine. We can speculate that gG3PD-mediated NBDHEX nitroreduction may be influenced by a redox-cycling property of 7-NBD derivative in presence of O_2_. NBF-SPh, a compound related to NBDHEX, has a redox-cycling activity with nitro group cycling to nitroso and hydroxylamine intermediates by reversible electron transfer to O_2_ before becoming exhausted and finally reduced to amine (Patridge et al., 2012). During redox-cycling, NBF-SPh rapidly converts O_2_ to ROS at an higher rate than that of the most toxic redox-cycling quinones and the nitrocompound MTZ. Bio-reduction of the redox cycling naphthoquinone menadione by flavoprotein/iron–sulphur-mediated electron transfer in both *G. duodenalis* trophozoites and cysts has been proven to be a potent generator of ROS, even in the presence of low levels of oxygen, thus leading to parasite killing (Paget et al., 2004). Therefore, we propose that the cytotoxicity exerted by NBDHEX against *G. duodenalis* could be due to its nitroreduction and to ROS generation during the reductive process, likely involving gG3PD. Finally, it should be considered that nitroreduction of NBDHEX, at least in part mediated by gG3PD, could subtract electrons from their physiological acceptor(s) thus altering the *G. duodenalis* intracellular redox metabolism.

Although further studies are necessary to fully understand the metabolic pathways involving the gG3PD, as well as the mechanisms of the enzyme regulation in *G. duodenalis*, we have clearly proven, for the first time, that a functional FAD-dependent gG3PD works in *G. duodenalis* and could be a valuable potential candidate for the design of novel antigiardial drugs having NBDHEX, a representative of novel class of antitumoral molecules, as leading compound.

## Footnotes

1 http://blast.ncbi.nlm.nih.gov

2 http://www.cbs.dtu.dk/services/TMHMM/

3 http://psort.hgc.jp/form2.html

4 http://ihg.gsf.de/ihg/mitoprot.html

## Supplementary material

TThe Supplementary Material for this article can be found online at: http://journal.frontiersin.org/article/10.3389/fmicb.2015.00544/abstract
