# Supplementary material for: The FAD-dependent glycerol-3-phosphate dehydrogenase of Giardia duodenalis: an unconventional enzyme that interacts with the g14-3-3 and it is a target of the antitumoral compound NBDHEX
Source: Front Microbiol. 2015 Jun 1;6:544. doi: 10.3389/fmicb.2015.00544 (PMC4450592; doi:10.3389/fmicb.2015.00544)
Supplement: Supplementary file 1 [file Image_1.PDF]

Supplemental Information

Supplemental Figure S1

A

MTTAHPLPIQSHTFFDSPQPFA**KLASAD**IY**DLAIIGGGISGVSAALEASHTTLKSILLERGLETSVATTRANSGI**VHCGV
HTTQGTLKAQLEQKGRRIFAHLCKSLNVSLRRCGELVIARNEAELNALQELQKSCAALDLECSLWGPARVKLEEPNLAID
SIEGALSIPETSVVNPFELCNAMANCAMANGVEISCGSTVTGIRKINRLAEEDGDALVESAPKVIFELRIS**RQGSSP**VYL
YAQTVINATGVDAGSVTELLKNATPINITKRRGELYVLDKNLKDVVT**RVIFSVI**PNNASSKGMLIIPTVDGTVMVGPTAE
PLGSTGSNMADIETTEDGRSTIFAHAHALCPRIVPEEIMYSFASARAVLPSNDFVIRDEDGFIQCAGIQSPGLTAGPAIG
AYLIELVKL**RYPSHR**MSSIVKPPEAWFWYPITSRLCRTALDKRHGLI**EKDLNFSKIVCGCECVTEADIENAITEGARSIT**
GLKLRTRATAGRCQGSFCSSKLRKLLAARLGVSYEQVCRISHN***TPMDKA***YPSSTVDVKLYRPLDCSEVGIRASLVSNLLF
GPKHTKESALNAFSLSTKLVQCIHAFDLVVLGTGPAGLGAALAAYDTNPDARILVITKDAYVGGSLMYALHRSHNAGDGS
TALTGPEFAVQLREDLMKTGVITILNTFILDSSYNTS**RKLFTVK**CMSANVGQLEISAKAVIFCLGSREKHRFNAAIPGTR
PYGVMTADAALRSIAFDNVLPGKRVVIFGSGDSGLLAARQISISGGQVLGIYEPESHLVGNSQYLDLLTKEFDIPISYN**K**
SISRINGQGVIESVCIAPVNPLTKGVTADEAETVACDCLVTSLGTLPQMALVKGMGCKGVSSQYCGLLVDNRCAVASDKL
LYAAGGNLHCHPSAESSFAEGKLAGFNAAIDVGDTARSYNPPSVNLTLSETNRSDIKYFMPQSITLSKRPTDKSGETFIY
FKTTRAMANVRVTVSTTSTGKVLATEKFREVSPTVVTRMVVSNSAFQDLVPGVNITLKLEFDPHEFSVK**VVDNRLLGVFS**
TAIPVLNGAKPHVIVKNKTPVPVDRMSSVLKELNATPAIQVEAPVSAGQTVLRNVAGQTDFVACENIPAMK

B

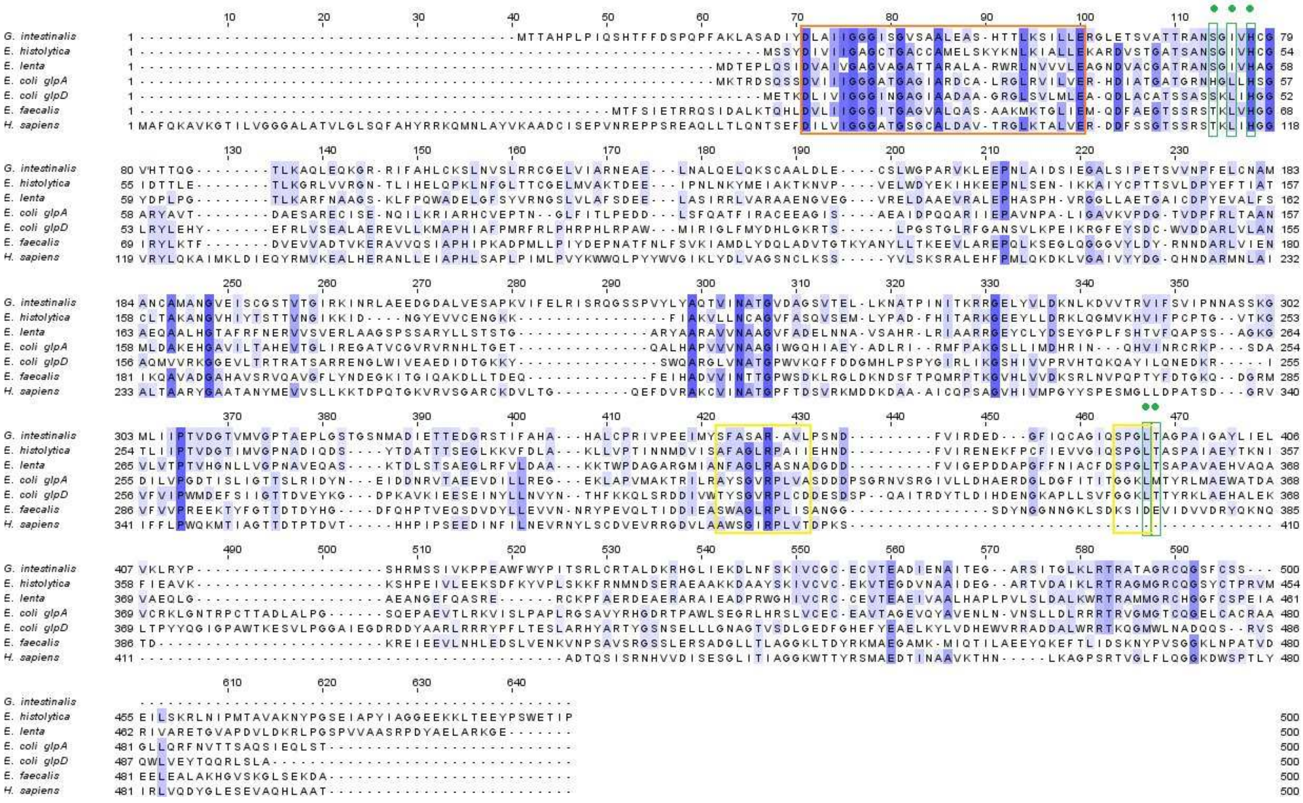

Supplemental Figure S1. A) Domain organization of the gG3PD protein. Light and dark green boxes, correspond to the multidomain TIGR03377 of subunit A (GlpA) protein family of FAD-dependent anaerobic glycerol-3-phosphate dehydrogenase (residues 31-510). A FAD-dependent oxidoreductase domain DAO (Pfam PF01266, residues 31-406) light green box and, a Bacterioferritin-associated ferredoxin (BFD)-like [2Fe-2S] binding domain (Fer2_BDF; PF04324, residues 455-510) dark green box. Black box, flavin binding domain (according to Cole et al., J Bacteriol. 1988 170(6):2448-56). Grey box corresponds to a small NADH binding domain within a larger FAD binding domain (Pyr_redox_2, PF07992, residues 587-910). Blue box correspond to a

domain found in hypothetical metal-binding proteins (DUF 1667, residues 587-910). Putative 14-3-3 binding site (according to Lalle et al., 2012) are in bold and underlined. Residues common to both the N- and C-terminal half constructs of the gG3PD are in bold and italic. **B) Multiple sequence alignment of the first portion of the G3PD from different species.** Multiple sequence alignment of the first portion (residues 1-510, including the glycerol-3-phosphate dehydrogenase-like domain) of Giardia duodenalis gG3PD (accession number: GL50803_16125) with the identified closer orthologues from Entamoeba histolytica (C4M6H3) and Eggerthella lenta (C8WGG6), and with the Escherichia coli anaerobic-glycerol-3-phosphate dehydrogenase subunit A (GlpA, P0A9C0), the E. coli aerobic glycerol-3-phosphate dehydrogenase (GlpD, P13035), the closely related glycerol-3-phosphate oxidase from Enterococcus faecalis (GlpO, F0PH56) and the human mitochondrial mG3PD (GPD2, P43304). Conserved residues above 30% of identity are highlighted in blue. Darker is the blue, higher is the degree of identity at the position. Orange box, Flavin Adenine Dinucleotide (FAD)-binding motif. Green box, residues that tightly interact (hydrogen bonding and hydrophobic interactions) with FAD and yellow box, residues potentially involved in glycerol-3-phosphate binding (according to Colussi, et al, 2008 and Yeh, et al, 2008).

Supplemental Figure S2

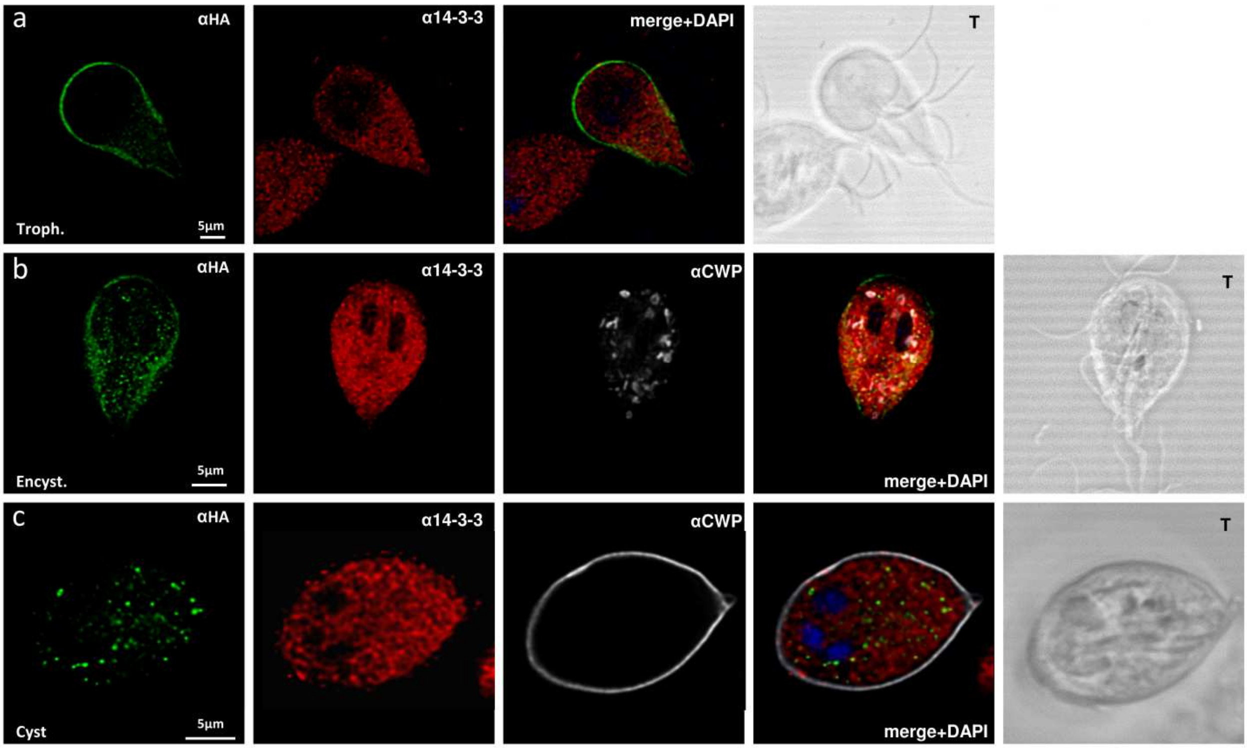

Supplemental Figure S2. Localization of the FLAG-HA-gG3PD during the differentiation stages of *G. duodenalis.* CLSM observations of fixed and permeabilized *G. duodenalis* WB-C6 transgenic parasites expressing the FLAG-HA-gG3PD protein. Trophozoite (panel a, Throph.), encysting parasite after 12h of encystation (panel b and c, Encyst.) and cyst (panel d, Cyst) stained with mouse α-HA mAb (green) and rabbit polyclonal α-g14-3-3 (red). Cyst wall and encystation specific vesicles (ESVs) were stained with Cy3-conjugated α-CWP mAb (grey). Nuclei were stained with DAPI (blue). Displayed micrographs correspond to a single z-stack. T, transmission light acquisition. Scale bars, 5µm.

Supplemental Figure S3

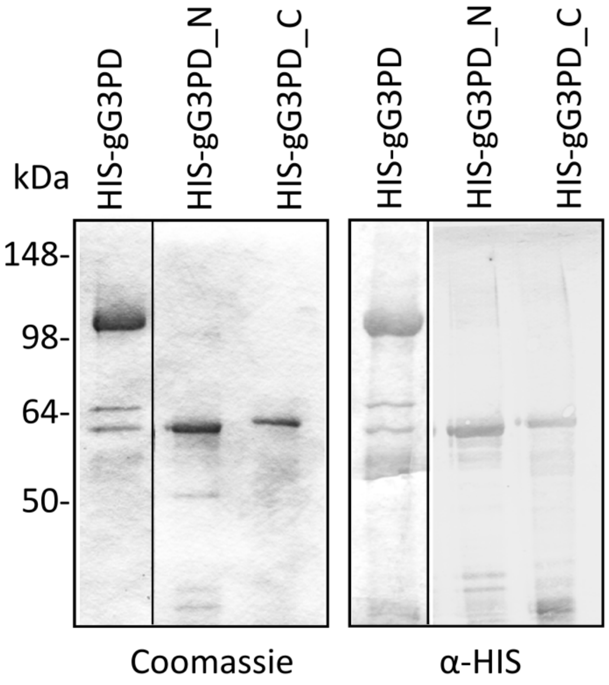

Supplemental Figure S3. Expression of recombinant HIS-gG3PD and deletion mutants in *Escherichia coli*. An aliquot of nickel-affinity purified recombinant HIS-gG3PD (2 mg), HIS-gG3PD_N (0.5 mg) and HIS-gG3PD_C (0.5 mg) were separated on 4-12% SDS-PAGE and either stained with Coomassie blue (Coomassie, left panel) or elettroblotted on nitrocellulose membrane and probed with mouse α-HIS mAb (right panel). Molecular size markers are on the left.

Supplemental Figure S4

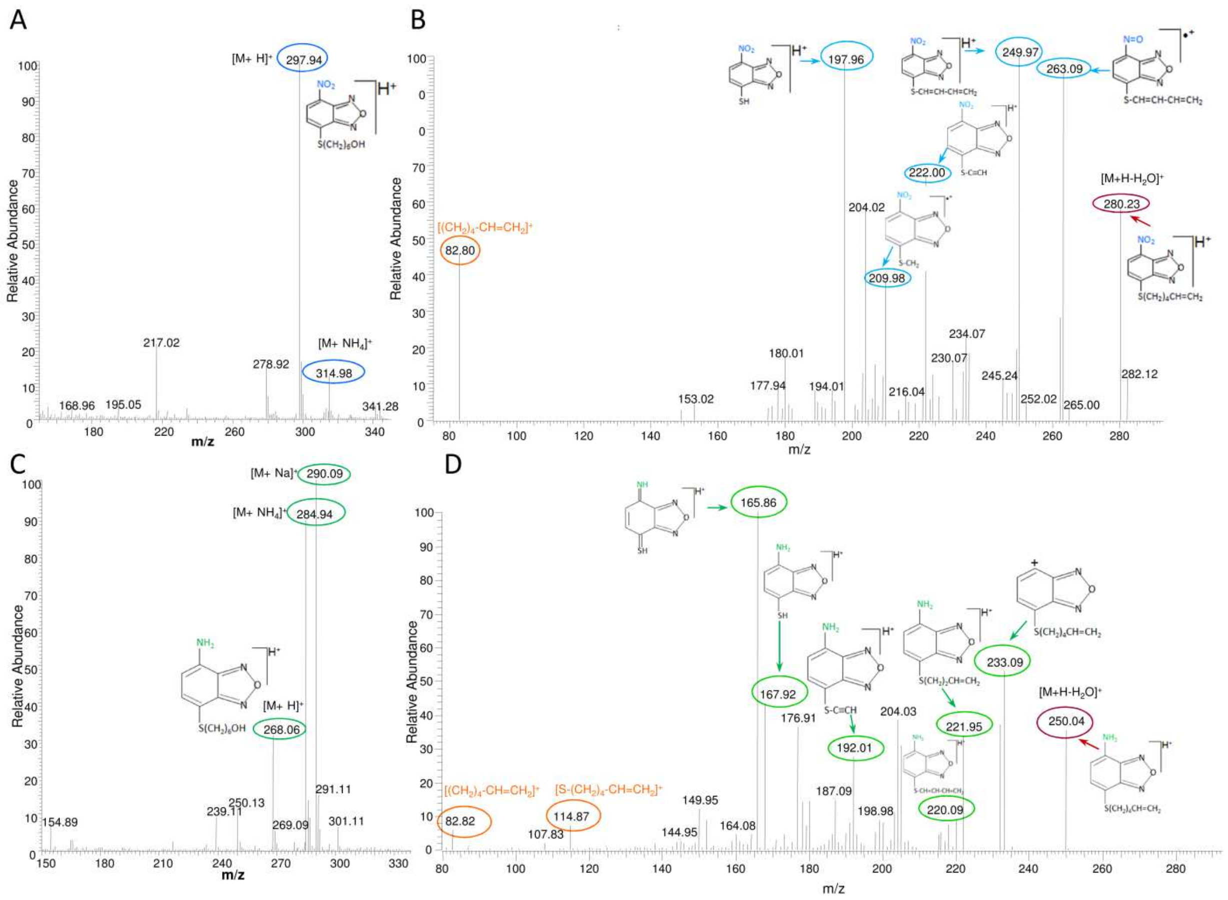

Supplemental Figure S4 NBDHEX reduction by sodium dithionite. Full MS of the untreated NBDHEX (A) or NBDHEX treated with sodium dithionite (C). MS/MS of the ions at m/z 298 and 268 (unmodified and reduced NBDHEX, respectively) give rise to neutral loss of a water molecule generating the ions at m/z 280 and 250, whose further fragmentation (MS3) is shown in panel B and D, respectively.

Supplemental Figure S5

A

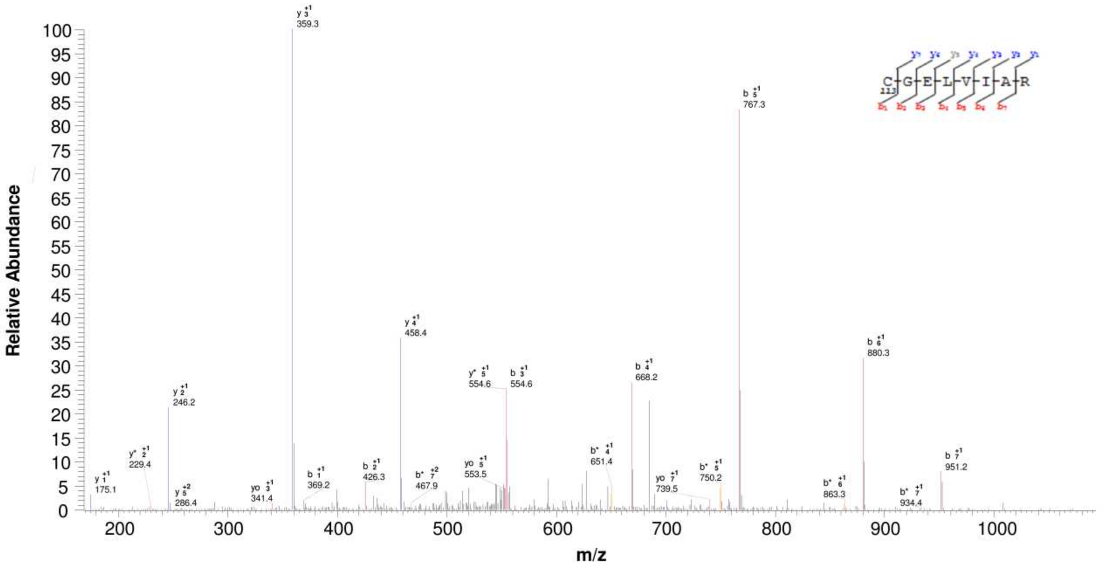

B

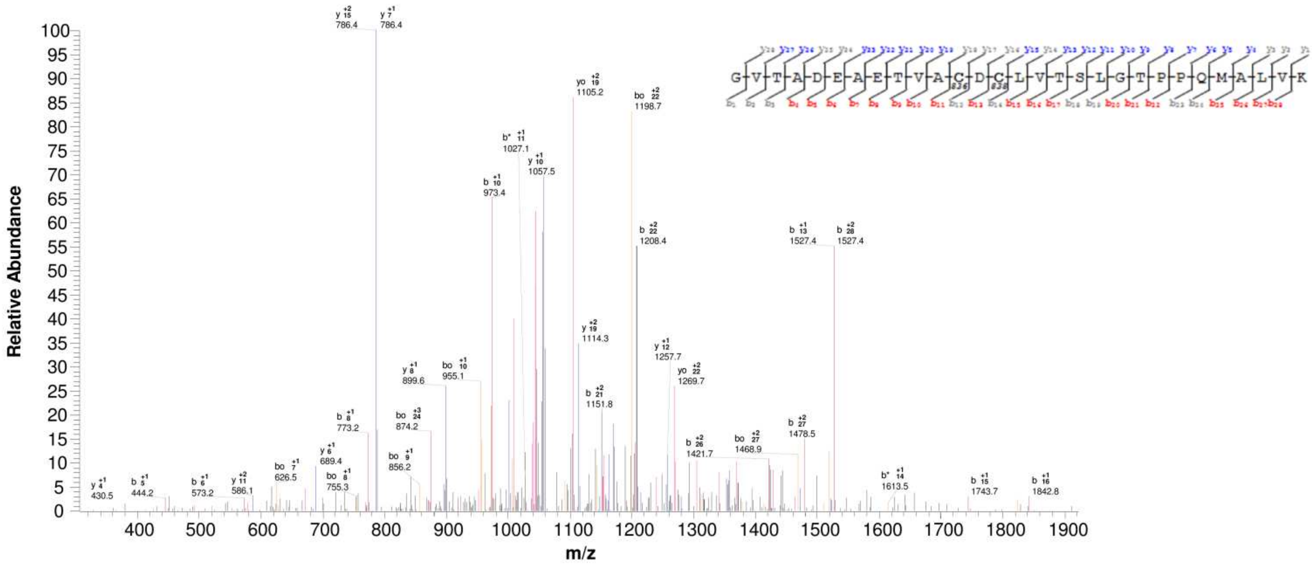

C

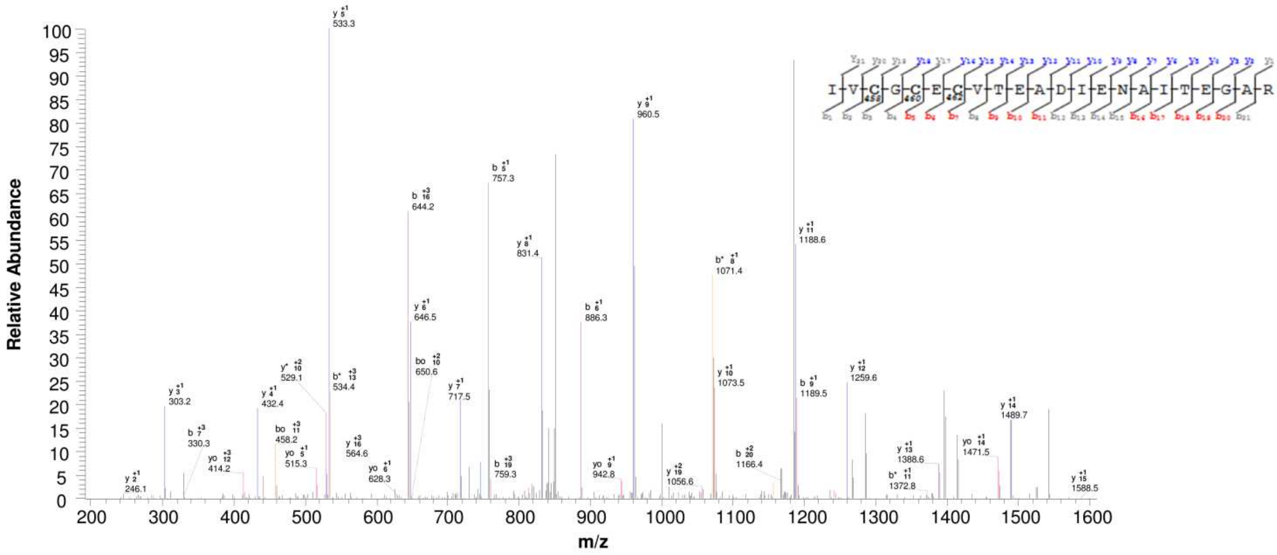

Supplemental Figure S5. MS analysis of the in vitro reaction between HIS-gG3PD and NBDHEX. MS/MS spectra matching the gG3PD peptides (113-120) (A), (859-872) (B), and (456-477) (C) carrying cysteine adducts with a mass increase of 265 Da (A and B) and 281 Da (C). The mass shift +265 Da is compatible with an NBDHEX derived adduct in which the nitro group was reduced to an amine one, while the mass increment of 281 Da matches with an NBHEX adduct in which the nitro group was semi-reduced to an hydroxyamine group. In the peptide (113-120) the unique cysteine residue (Cys133) is involved in the adduct as clearly demonstrated by the fragment pattern detected in the MS/MS spectrum (A). The peptide (859-872) contains two cysteine residues (Cys836 and Cys838) and the adduct is more probably located on the first cysteine residue (B). In the peptide (456-477) there are three candidate cysteine residues at positions 458, 460, and 462: the Cys 458 is most likely the one involved in the adduct formation, but the involvement of the second or the third cysteine cannot be excluded (C).

References

1. Colussi, T., Parsonage, D., Boles, W., Matsuoka, T., Mallett, T.C., Karplus, P.A., *et al.* (2008). Structure of alpha-glycerophosphate oxidase from Streptococcus sp.: a template for the mitochondrial alpha-glycerophosphate dehydrogenase. *Biochemistry*. 47, 965-977.
